# Triphala Targets the SLC7A11–GSH–GPX4 Axis to Trigger Ferroptosis in Oral Cancer: An Integrated Network Pharmacology, Molecular Docking, and Experimental Validation Study

**DOI:** 10.1111/cbdd.70375

**Published:** 2026-08-26

**Authors:** Yiwei Zhao, Simin Li, Linxin Jiang, Deborah Kreher, Gerhard Schmalz, Andreas Fichter, Xianda Hu

**Affiliations:** ^1^ Department of Craniomaxillofacial Surgery Leipzig University Leipzig Germany; ^2^ Laboratory of Molecular Cell Biology Beijing Tibetan Hospital, China Tibetology Research Center Beijing China; ^3^ Department of Prosthodontics University of Leipzig Leipzig Germany; ^4^ Department of Conservative Dentistry and Periodontology Brandenburg Medical School (MHB) Theodor Fontane Brandenburg an der Havel Germany

**Keywords:** ferroptosis, mendelian randomization, network pharmacology, oral cancer, prognostic signature, SLC7A11–GSH–GPX4 axis, Triphala

## Abstract

Triphala is a traditional three‐fruit formulation with potential anticancer activity, but its ferroptosis‐related mechanisms in oral cancer remain unclear. We integrated network pharmacology, transcriptomic analyses, prognostic modeling, Mendelian randomization, immune and drug‐response analyses, molecular docking, and in vitro validation to investigate the Triphala–ferroptosis–oral cancer axis. Fifty‐eight candidate functional genes were identified, and an eight‐gene signature comprising AKR1C3, CA9, EGFR, GSTA1, MAPK8, MGST1, PPARG, and RB1 showed prognostic value across multiple cohorts. Mendelian randomization supported causal associations of MAPK8, MGST1, and PPARG with oral cancer risk. Seven Triphala‐derived compounds, including epigallocatechin gallate, quercetin, kaempferol, luteolin, ellagic acid, gallic acid, and quinine, displayed favorable predicted interactions with key targets. In CAL‐27 cells, Triphala altered the expression of signature genes, reduced GPX4 and SLC7A11 protein levels, increased Fe^2+^and malondialdehyde, depleted glutathione and glutathione peroxidase activity, and enhanced lipid peroxidation; these effects were partially modulated by ferrostatin‐1. This study advances the field by linking Triphala to a ferroptosis‐based prognostic framework and experimentally demonstrating its regulation of the SLC7A11–GSH–GPX4 axis in oral cancer.

## Graphical abstract

Triphala targets ferroptosis‐related pathways in oral cancer through multi‐compound, multi‐target interactions identified by network pharmacology and molecular docking. In CAL‐27 cells, Triphala suppresses the GPX4/SLC7A11 axis, promotes iron accumulation and lipid peroxidation, and supports an eight‐gene prognostic signature.

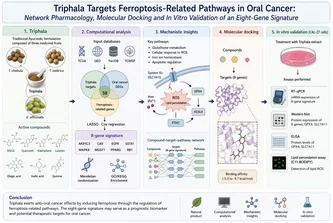

## 1 Introduction

Oral cancer represents a significant global health burden with increasing incidence and mortality rates worldwide. Triphala, a traditional herbal formulation composed of three fruits—*Emblica officinalis, Terminalia belerica*, and *Terminalia chebula* —has emerged as a promising complementary therapeutic agent for oral cancer management due to its diverse bioactive compounds and pharmacological properties (Sandhya et al. 2006). This ancient formulation exhibits potent antioxidant, anti‐inflammatory, immunomodulatory, and anticancer activities through complex molecular mechanisms (Kumar et al. 2016). Triphala has been demonstrated to inhibit oral squamous cell carcinoma (OSCC) progression by inactivating the PI3K/Akt signaling pathway, thereby reducing cellular metabolic activity, migration (Zhao et al. 2026), invasion, and proliferation while promoting apoptotic cell death (Hu et al. 2024). Additionally, Triphala modulates multiple signaling pathways including ERK, MAPK, and NF‐κB, which are crucial for cancer cell survival, and induces both intrinsic and extrinsic apoptotic pathways to control tumor growth (Zhao et al. 2018). The therapeutic potential of Triphala in oral cancer is further enhanced by its immunomodulatory properties, which strengthen the body's natural defense mechanisms against cancer cells through anti‐proliferative and tumor‐suppressive actions (Dineshkumar et al. 2024). Despite these promising attributes, the precise molecular mechanisms underlying Triphala's effects on oral cancer remain incompletely understood, necessitating further investigation to optimize its clinical application.

Ferroptosis, a relatively newly discovered form of regulated cell death, has gained significant attention in cancer research due to its unique iron‐dependent mechanism distinct from apoptosis and other cell death pathways. Unlike apoptosis, ferroptosis is characterized by the accumulation of iron, lipid peroxides, and reactive oxygen species (ROS), leading to oxidative damage of cellular membranes and subsequent cell death (Wang, Huang, et al. 2023). This iron‐dependent cell death pathway offers promising therapeutic potential, particularly for malignancies resistant to conventional treatments that typically target apoptotic mechanisms (Guo et al. 2023). Herbal medicines, especially those from traditional medical systems, have demonstrated considerable potential in modulating ferroptotic pathways. These natural compounds can influence ferroptosis through multiple mechanisms, including disruption of iron metabolism, inhibition of glutathione peroxidase 4 (GPX4), downregulation of system xc‐ (cystine/glutamate antiporter), and enhancement of lipid peroxidation (Wu et al. 2022). For instance, quercetin exhibits iron‐chelating properties that can induce ferroptosis in cancer cells (Yin et al. 2021), while curcumin and resveratrol modulate multiple components of the tumor microenvironment to enhance ferroptotic cell death (Park et al. 2021). Various traditional Chinese herbal formulations containing *Salvia miltiorrhiza* and flavonoids have demonstrated the ability to regulate iron overload and lipid peroxidation, contributing to ferroptosis in cancer cells (Liu et al. 2024). Despite these promising findings, there remains a significant research gap in understanding the specific molecular mechanisms through which traditional herbal formulations like Triphala might influence ferroptosis in oral cancer. Notably, previous investigations of Triphala in OSCC have focused almost exclusively on apoptosis‐centered mechanisms. Hu et al. combined network pharmacology, molecular docking, and in vitro and zebrafish‐xenograft validation to show that Triphala suppresses OSCC proliferation, migration, and invasion and promotes apoptosis by inactivating the PI3K/Akt signaling pathway through hub genes such as JUN, EGFR, ESR1 (Guo et al. 2025), RELA, and AKT1 (Hu et al. 2024). Likewise, a recent systematic review concluded that the anticancer activity of Triphala in oral cancer and oral precancer is mediated chiefly by anti‐oxidative, anti‐proliferative, immunomodulatory, and apoptotic actions (Dineshkumar et al. 2024). However, whether Triphala exerts its antitumor effects in oral cancer through ferroptosis—an iron‐dependent, lipid‐peroxidation‐driven form of cell death mechanistically distinct from apoptosis—has not been examined, and no prior study has linked Triphala to a ferroptosis‐based prognostic signature or established causal target genes in this context. The present study addresses this gap by shifting the mechanistic focus from apoptosis to ferroptosis and, for the first time, integrating multi‐omics evidence with experimental validation to define how Triphala engages the ferroptosis machinery in oral cancer.

**FIGURE 1:**
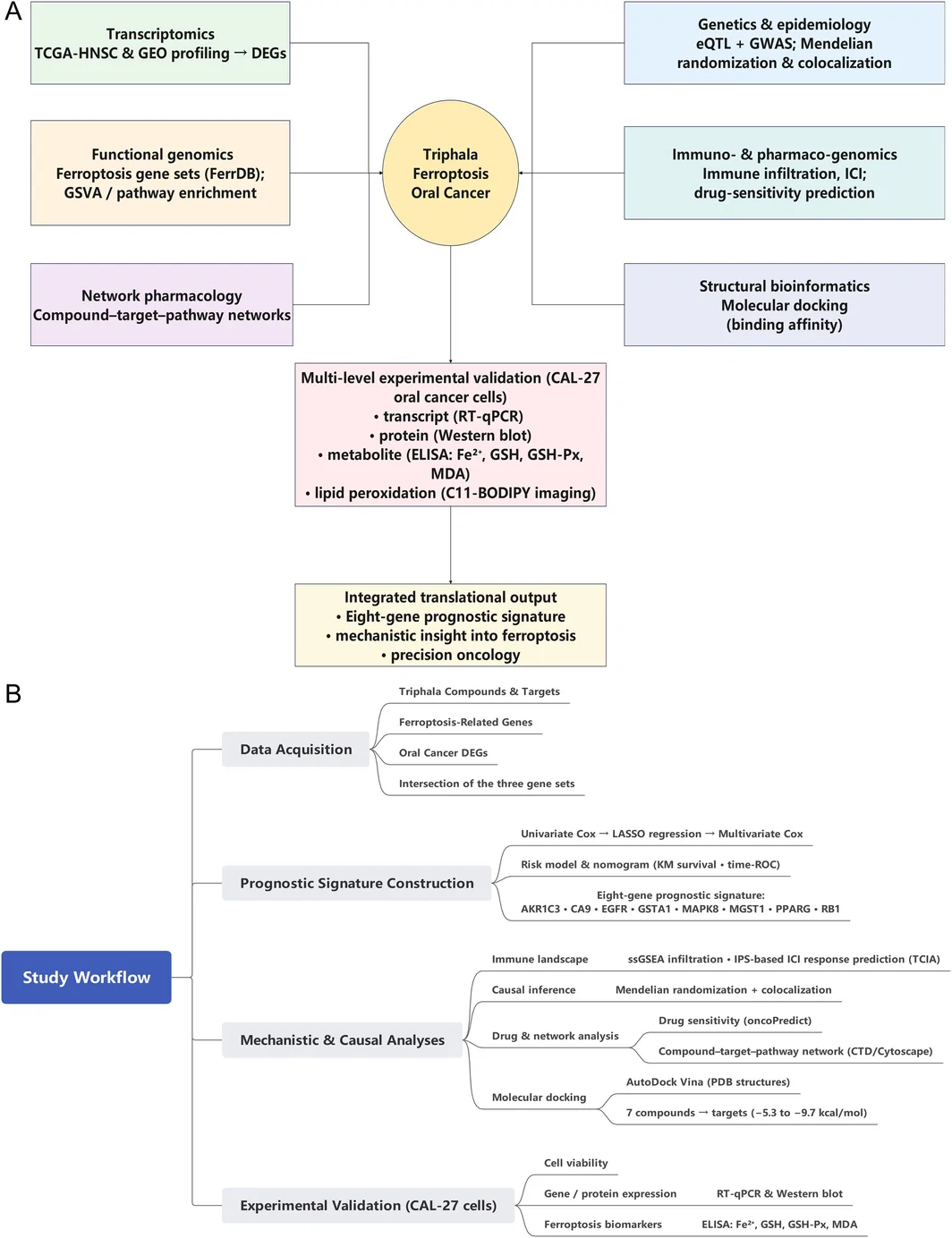
Overview of the integrative multi‐omics framework and analytical workflow of the study. (A) Integrative multi‐omics framework converging on the Triphala–ferroptosis–oral cancer axis. Six complementary omics and computational layers—transcriptomics (TCGA‐HNSC and GEO expression profiling), functional genomics (ferroptosis‐related gene curation and pathway enrichment), network pharmacology (compound–target–pathway modeling), genetics and genetic epidemiology (eQTL‐ and GWAS‐based Mendelian randomization with colocalization), immuno‐ and pharmacogenomics (immune infiltration, immune checkpoint inhibitor response, and drug‐sensitivity prediction), and structural bioinformatics (molecular docking)—are integrated and subsequently cross‐validated by multi‐level experimental assays at the transcript, protein, metabolite, and lipid levels, yielding an eight‐gene prognostic signature and mechanistic insight for precision oncology. (B) Schematic workflow illustrating the sequential analytical steps of the study, from data acquisition (Triphala targets, ferroptosis‐related genes, and oral cancer differentially expressed genes) through functional gene identification, prognostic signature construction, mechanistic and causal analyses, and in vitro experimental validation, culminating in the proposed model of Triphala‐induced ferroptosis via suppression of the SLC7A11–GSH–GPX4 axis.

In recent years, the integration of multi‐omics technologies has become a powerful strategy for dissecting the molecular complexity of cancer, allowing transcriptomic, genetic, pharmacological, and structural information to be combined into a unified mechanistic picture. Such multidimensional omics approaches have proven particularly valuable for resolving the heterogeneity of the tumor immune microenvironment and the mechanisms underlying resistance to immunotherapy (Li et al. 2025; Yin et al. 2025). Building on this paradigm, the present study employs an integrative multi‐omics framework that converges transcriptomics, functional genomics, network pharmacology, genetic epidemiology, immuno‐ and pharmacogenomics, and structural bioinformatics on the Triphala–ferroptosis–oral cancer axis, followed by multi‐level experimental validation at the transcript, protein, metabolite, and lipid levels (Figure 1A).

The present study aims to elucidate the molecular mechanisms by which Triphala modulates ferroptosis in oral cancer through an integrated network pharmacology–bioinformatics–experimental validation framework. Specifically, we identified bioactive compounds in Triphala and predicted their putative targets, intersected these targets with ferroptosis‐related genes and oral cancer differentially expressed genes (DEGs), and constructed a prognostic gene signature. We then performed multi‐layered analyses—including gene set variation analysis (GSVA), Mendelian randomization, immune infiltration evaluation, drug sensitivity prediction, and molecular docking—to prioritize key functional genes, signaling pathways, and candidate bioactive compounds underlying Triphala's ferroptosis‐related antitumor effects. Importantly, these computational findings were further validated experimentally in oral cancer cells: Triphala treatment was assessed using cell viability assays and ferroptosis‐focused readouts, and the expression of signature genes and canonical ferroptosis markers was confirmed by RT‐qPCR, Western blotting, ELISA‐based measurements of ferroptosis‐related biomarkers, and lipid peroxidation assays. Together, this multifaceted strategy provides mechanistic insight into Triphala‐induced ferroptosis in oral cancer while highlighting potential prognostic biomarkers and therapeutic targets for future translational development.

## 2 Methods

### 2.1 Study Design and Analytical Workflow

The overall design of this study, integrating network pharmacology (Li et al. 2023), bioinformatics, causal inference, and experimental validation, is summarized in Figure 1B. First, we assembled three complementary gene sets: the putative targets of Triphala's bioactive compounds, retrieved from the TCMSP and TCM Database@Taiwan and filtered by oral bioavailability (OB ≥ 30%) and drug‐likeness (DL ≥ 0.18); ferroptosis‐related genes obtained from the FerrDB V2 database; and DEGs in oral cancer, identified from the TCGA‐HNSC discovery cohort and validated across four independent GEO datasets. Intersecting these three sets yielded a panel of functional genes that were subsequently characterized by Metascape enrichment analysis and GSVA. We then constructed a prognostic model through sequential univariate Cox, LASSO, and multivariate Cox regression, deriving an eight‐gene signature (AKR1C3, CA9, EGFR, GSTA1, MAPK8, MGST1, PPARG, and RB1) that was evaluated using risk stratification, Kaplan–Meier survival analysis, time‐dependent ROC curves, and a nomogram. To dissect the underlying mechanisms, we performed a series of parallel analyses, including immune infiltration and immune checkpoint inhibitor response prediction (ssGSEA and IPS), Mendelian randomization with colocalization to establish causal relationships, drug sensitivity analysis (oncoPredict), compound–target–pathway network construction, and molecular docking. Finally, the key computational findings were experimentally validated in CAL‐27 oral cancer cells using CCK‐8 viability assays, RT‐qPCR, Western blotting, ELISA‐based ferroptosis biomarker quantification (Fe^2+^, GSH, GSH‐Px, and MDA), and C11‐BODIPY lipid peroxidation imaging. The detailed procedures for each step are described in the following sections.

### 2.2 Acquisition and Analysis of Small Molecules in Triphala

Triphala is a polyherbal formulation composed of the dried fruits of three plants in equal proportions: *Terminalia chebula* (Hezi), *Terminalia bellirica* (Maohezi), and *Phyllanthus emblica* (syn. *Emblica officinalis*; Yuganzi). The chemical constituents of each herb were retrieved from the Traditional Chinese Medicine Systems Pharmacology Database (TCMSP; https://tcmsp‐e.com/) and the TCM Database@Taiwan, and the records were merged and de‐duplicated by compound name and molecule ID. To prioritize orally active, drug‐like constituents, candidates were screened using two ADME parameters provided by TCMSP: OB and DL, retaining molecules with OB ≥ 30% and DL ≥ 0.18 as primary bioactive compounds. The putative targets of the selected compounds were then predicted in TCMSP, and the corresponding protein names were converted to official human gene symbols ( *Homo sapiens* ) using the UniProt database.

### 2.3 Oral Cancer Data Collection and Processing

Transcriptomic and clinical data from oral cancer patients were acquired from The Cancer Genome Atlas (TCGA‐HNSC) and Gene Expression Omnibus (GEO) databases. From TCGA, samples labeled 01A were designated as the disease group and 11A as the control group. Oral cancer samples were identified based on anatomical sites including oral tongue, floor of mouth, buccal mucosa, alveolar ridge, hard palate, and oral cavity. For validation, additional datasets (GSE30784, GSE41613, GSE42743, GSE65858) were utilized, with TCGA‐HNSC serving as the primary analysis dataset and others for validation.

### 2.4 Ferroptosis Gene Identification

Ferroptosis‐related genes were downloaded from the ferrdb V2 database (http://www.zhounan.org/ferrdb/current/), which contains 621 ferroptosis‐associated genes categorized as drivers (264), suppressors (238), markers (9), and unclassified genes (110). After removing duplicates, 564 unique ferroptosis genes were identified for subsequent analysis.

### 2.5 Data Preprocessing and Differential Expression Analysis

For comprehensive assessment of oral cancer gene expression profiles, we utilized multiple datasets as detailed in Table 1. These datasets were selected because they provide transcriptomic profiles of oral squamous cell carcinoma and, where available, matched clinical survival information, enabling both robust differential expression analysis and prognostic modeling. The primary analysis dataset was The Cancer Genome Atlas head and neck squamous cell carcinoma cohort (TCGA‐HNSC), which provided high‐throughput sequencing data from 309 tumor samples and 30 normal tissues, along with comprehensive survival information; owing to its large sample size and paired transcriptomic and survival data, TCGA‐HNSC served as the discovery cohort for differential expression analysis and prognostic signature construction. To validate our findings, we incorporated four independent Gene Expression Omnibus (GEO) datasets profiled on different microarray platforms. GSE30784 (Affymetrix GPL570 platform) contained 167 oral cancer cases and 45 normal controls but lacked survival data. GSE41613 (GPL570 platform) included 76 cases with survival information but no normal controls. GSE42743 (GPL570 platform) provided balanced data with 55 tumor samples, 29 normal tissues, and corresponding survival records. Finally, GSE65858 (Illumina GPL10558 platform) contributed 83 additional oral cancer cases with survival data. This multi‐dataset approach ensured robust identification of molecular signatures across diverse patient populations and technical platforms, with TCGA‐HNSC serving as the discovery cohort and the GEO datasets providing independent validation. Datasets with available survival information (marked as “Y”) were particularly valuable for prognostic marker identification and survival analysis, while those without survival data (marked as “N”) contributed to the identification of DEGs between tumor and normal tissues.

**TABLE 1:** Datasets used for oral cancer analysis. Summary of the five datasets used in this study, including their identifiers, platforms, sample sizes for case and control groups, and availability of survival data (Y: available, N: not available).

Dataset ID	Platform	Case	Normal	Survival data
TCGA‐HNSC	High‐throughput sequencing	309	30	Y
GSE30784	GPL570	167	45	N
GSE41613	GPL570	76	—	Y
GSE42743	GPL570	55	29	Y
GSE65858	GPL10558	83		Y

### 2.6 Functional Gene Identification and Enrichment Analysis

To explore relationships between Triphala, ferroptosis, and oral cancer, we identified functional genes by taking the union of pairwise intersections between Triphala targets, ferroptosis genes, and DEGs in oral cancer. In this study, “functional genes” were therefore defined as the genes shared by at least two of these three datasets, representing candidate genes through which Triphala may exert ferroptosis‐related effects in oral cancer. Functional enrichment analysis was performed using Metascape (https://metascape.org/) to identify significantly enriched pathways and biological processes associated with these functional genes.

### 2.7 Gene Set Variation Analysis

Expression values of Triphala targets, ferroptosis genes, and identified functional genes were extracted from oral cancer samples for GSVA analysis using the “GSVA” package in R. Differential pathway analysis was performed using the “limma” package to identify pathways with significant differences between disease and control groups.

### 2.8 Functional Gene Selection and Prognostic Model Construction

Univariate Cox proportional hazards regression analysis was performed to identify genes significantly associated with patient survival (*p* < 0.05). LASSO (Least Absolute Shrinkage and Selection Operator) regression was then applied to select the most important prognostic genes. Multivariate Cox‐PH analysis was conducted on these selected genes to construct a risk score model. Patients were stratified into high and low‐risk groups based on the median risk score, and survival analyses at 3, 5, and 10 years were performed to evaluate prognostic differences.

### 2.9 Immune Cell Proportion Score and Immune Infiltration Analysis

Immune Cell Proportion Scores (IPS) were obtained from The Cancer Immunome Atlas (TCIA) database to predict patient response to immune checkpoint inhibitors. Single‐sample Gene Set Enrichment Analysis (ssGSEA) was employed to assess the relative abundance of 28 immune cell subpopulations across all datasets. Pearson correlation analysis was used to identify significant relationships (*p* < 0.05) between functional genes and immune cell populations.

### 2.10 Mendelian Randomization and Colocalization Analysis

Causal relationships between functional genes and oral cancer were investigated using Mendelian randomization. Gene coordinates in hg19 were obtained from NCBI Gene, and eQTL data were retrieved from eQTLGen. Oral cavity cancer GWAS data (dataset ieu‐b‐4961, *n* = 372,373) were accessed from OpenGWAS. Significant (*p* < 5 × 10^−8^) and linkage‐balanced SNPs were selected as instrumental variables. Causal effects were analyzed using various methods including inverse‐variance weighted, MR‐Egger, weighted median, simple mode, and weighted mode. Colocalization analysis was performed using the “coloc” package to identify potential functional variants.

### 2.11 Small Molecule Analysis and Drug Sensitivity

Triphala small molecules corresponding to functional genes were extracted and their biological functions were obtained from the Comparative Toxicogenomics Database (CTD). Compound‐drug target‐pathway networks were constructed using Cytoscape to analyze ferroptosis‐related functions regulated by compounds and drug targets. Drug sensitivity analysis was performed using the “oncoPredict” package to identify drugs highly correlated with functional genes through Pearson correlation analysis.

### 2.12 Molecular Docking Simulation

To validate the interactions between Triphala‐derived bioactive compounds and the identified functional genes, molecular docking simulations were performed using three‐dimensional structures of target proteins retrieved from the Protein Data Bank (PDB) with the following PDB IDs: AKR1C3 (4DBS), EGFR (1WT5), GSTA1 (7BIB), MAPK8 (4G1W), MGST1 (6VL4), PPARG (9CK0), and RB1 (9MML). The chemical structures of Triphala bioactive compounds (kaempferol, luteolin, quinine, quercetin, epigallocatechin gallate, ellagic acid, and gallic acid) were obtained from the PubChem database and optimized using molecular modeling software, while all protein structures were prepared by removing water molecules, adding hydrogen atoms, and optimizing side chain conformations with binding sites identified based on known functional domains and literature reports. Molecular docking was performed using AutoDock Vina with default parameters, selecting the top‐ranked binding poses with the most favorable binding energies (lowest kcal/mol values) for analysis, and binding interactions were visualized to identify key residues involved in compound‐protein interactions, including hydrogen bonds, hydrophobic interactions, and π–π stacking interactions.

### 2.13 Preparation of Triphala Extract

Triphala extract was prepared from a commercially available Triphala powder (Dabur India Ltd., Alwar, India; batch number: AL1675) containing an equal proportion (1:1:1) of three botanical components: *Terminalia chebula* , *Emblica officinalis*, and *Terminalia bellirica* . The extraction procedure was performed as follows: finely powdered Triphala was subjected to reflux extraction using ultrapure water at a material‐to‐solvent ratio of 1:10 (w/v) for 1 h. Following extraction, the mixture was centrifuged at 2860 × *g* for 15 min, and the supernatant was collected and filtered through a 0.45‐μm membrane filter (Merck Millipore Ltd., Cork, Ireland) to remove particulate matter. The filtered extract was then concentrated under reduced pressure using a rotary evaporator, followed by lyophilization to obtain a dry extract powder. The resulting yellowish‐green powder was weighed, aliquoted, and stored at −20°C until further use. For cell culture experiments, the lyophilized extract was reconstituted in culture medium to achieve the desired working concentrations.

### 2.14 CCK‐8 Cell Viability Assay

Cell viability was assessed using the Cell Counting Kit‐8 (CCK‐8) assay according to the manufacturer's instructions. CAL‐27 cells were seeded in 96‐well plates at a density of 5 × 10^3^ cells per well and allowed to adhere overnight. Cells were then treated with Triphala extract at various concentrations (0, 20, 40, and 60 μg/mL) for 24 h. Following treatment, 10 μL of CCK‐8 solution was added to each well and incubated for 2 h at 37°C in a humidified atmosphere containing 5% CO_2_. The absorbance was measured at 450 nm using a microplate reader. Cell viability was calculated as a percentage relative to the untreated control group. Each experiment was performed in triplicate and repeated at least three times independently.

### 2.15 Quantitative Real‐Time PCR

To examine the expression of ferroptosis‐related genes at the mRNA level, CAL‐27 cells were treated with vehicle (control) or 40 μg/mL Triphala extract for 24 h. Total RNA was extracted using TRIzol reagent (Invitrogen, Carlsbad, CA, USA) according to the manufacturer's protocol. RNA concentration and purity were determined using a NanoDrop spectrophotometer (Thermo Fisher Scientific, Waltham, MA, USA), with A260/A280 ratios between 1.8 and 2.0 considered acceptable. One microgram of total RNA was reverse transcribed into complementary DNA (cDNA) using the PrimeScript RT Reagent Kit (Takara, Dalian, China) following the manufacturer's instructions.

Quantitative real‐time PCR was performed using SYBR Green PCR Master Mix (Takara, Dalian, China) on a QuantStudio 6 Flex Real‐Time PCR System (Applied Biosystems, Foster City, CA, USA). The PCR reaction mixture contained 10 μL of SYBR Green Master Mix, 0.5 μL of each primer (10 μM), 2 μL of cDNA template, and nuclease‐free water to a final volume of 20 μL. The thermal cycling conditions were as follows: initial denaturation at 95°C for 30 s, followed by 40 cycles of denaturation at 95°C for 5 s and annealing/extension at 60°C for 30 s. Melting curve analysis was performed to verify the specificity of the PCR products.

The expression levels of eight ferroptosis‐related genes (AKR1C3, CA9, EGFR, GSTA1, MAPK8, MGST1, PPARG, and RB1) were analyzed. GAPDH was used as an internal reference gene for normalization. The relative mRNA expression levels were calculated using the 2^(‐ΔΔCt) method. Each sample was analyzed in triplicate, and experiments were repeated at least three times independently.

### 2.16 Western Blot Assay

To evaluate the protein expression levels of ferroptosis‐related genes, CAL‐27 cells were seeded in 6‐well plates and allowed to adhere overnight. Cells were then treated with vehicle (control), 40 μg/mL Triphala extract, 1 μM Fer − 1, or a combination of 40 μg/mL Triphala extract and 1 μM Fer‐1 for 24 h. After treatment, cells were harvested and lysed with RIPA lysis buffer (Beyotime Biotechnology, Shanghai, China) containing protease inhibitor cocktail (Roche, Basel, Switzerland) and phosphatase inhibitors (Sigma‐Aldrich, St. Louis, MO, USA) on ice for 30 min. Cell lysates were centrifuged at 12,000 × *g* for 15 min at 4°C, and the supernatants were collected. Protein concentrations were determined using the BCA Protein Assay Kit (Thermo Fisher Scientific, Waltham, MA, USA).

Equal amounts of protein (30–50 μg) were separated by 10% or 12% sodium dodecyl sulfate‐polyacrylamide gel electrophoresis (SDS‐PAGE) and subsequently transferred onto polyvinylidene fluoride (PVDF) membranes (Millipore, Bedford, MA, USA). The membranes were blocked with 5% non‐fat milk in Tris‐buffered saline containing 0.1% Tween‐20 (TBST) for 1 h at room temperature. After blocking, the membranes were incubated overnight at 4°C with primary antibodies against AKR1C3 (1:1000, Cell Signaling Technology, Danvers, MA, USA), CA9 (1:1000, Abcam, Cambridge, UK), EGFR (1:1000, Cell Signaling Technology), GSTA1 (1:1000, Proteintech, Rosemont, IL, USA), MAPK8 (1:1000, Cell Signaling Technology), MGST1 (1:1000, Abcam), PPARG (1:1000, Cell Signaling Technology), RB1 (1:1000, Cell Signaling Technology), GPX4 (1:1000, Abcam), SLC7A11 (1:1000, Cell Signaling Technology), and GAPDH (1:5000, Proteintech) as a loading control.

Following primary antibody incubation, membranes were washed three times with TBST and then incubated with horseradish peroxidase (HRP)‐conjugated secondary antibodies (1:5000, Cell Signaling Technology) for 1 h at room temperature. After three additional washes with TBST, protein bands were visualized using enhanced chemiluminescence (ECL) reagent (Thermo Fisher Scientific) and detected with a ChemiDoc imaging system (Bio‐Rad, Hercules, CA, USA). Band intensities were quantified using ImageJ software (National Institutes of Health, Bethesda, MD, USA), and protein expression levels were normalized to GAPDH. Each experiment was repeated at least three times independently.

### 2.17 ELISA Detection of Ferroptosis‐Related Biomarkers

To investigate whether Triphala‐induced cell death occurs through the ferroptosis pathway, CAL‐27 cells were seeded in 6‐well plates at a density of 2 × 10^5^ cells per well and allowed to adhere overnight. Cells were then divided into four experimental groups: (1) Control group: cells treated with vehicle (DMSO); (2) Triphala group: cells treated with 40 μg/mL Triphala extract; (3) Ferrostatin‐1 (Fer‐1) group: cells treated with 1 μM Fer‐1 (a specific ferroptosis inhibitor, Sigma‐Aldrich, St. Louis, MO, USA); and (4) Rescue group: cells co‐treated with 40 μg/mL Triphala extract and 1 μM Fer‐1. After 24 h of treatment, cells were harvested and washed twice with ice‐cold PBS.

For ELISA analysis, cell pellets were lysed using RIPA lysis buffer containing protease inhibitors on ice for 30 min, followed by centrifugation at 12,000 × *g* for 15 min at 4°C to collect the supernatant. Protein concentrations were determined using the BCA Protein Assay Kit (Thermo Fisher Scientific, Waltham, MA, USA). The levels of ferroptosis‐related biomarkers were quantified using enzyme‐linked immunosorbent assay (ELISA) kits according to the manufacturers' instructions: intracellular ferrous iron (Fe^2+^) levels were measured using the Fe^2+^ ELISA Kit (Elabscience, Wuhan, China), reduced glutathione (GSH) content was detected using the GSH ELISA Kit (Elabscience, Wuhan, China), glutathione peroxidase 4 (GSH‐Px) activity was assessed using the GSH‐Px ELISA Kit (Elabscience, Wuhan, China), and malondialdehyde (MDA) levels were determined using the MDA ELISA Kit (Elabscience, Wuhan, China). Briefly, samples and standards were added to pre‐coated 96‐well plates and incubated according to the kit protocols. After washing, detection antibodies and substrate solutions were added sequentially. The reaction was stopped, and absorbance was measured at 450 nm using a microplate reader (BioTek, Winooski, VT, USA). All samples were analyzed in triplicate, and concentrations were calculated based on standard curves.

### 2.18 Lipid Peroxidation Detection by C11‐BODIPY Staining

To visualize lipid peroxidation in live cells, CAL‐27 cells were seeded in confocal dishes and treated with different conditions as described above for 24 h. Lipid peroxidation was detected using the C11‐BODIPY 581/591 probe (Thermo Fisher Scientific, Waltham, MA, USA), a fluorescent lipid peroxidation sensor. Following treatment, cells were incubated with 2 μM C11‐BODIPY probe diluted in serum‐free medium for 30 min at 37°C in the dark. After incubation, cells were washed three times with PBS to remove excess probe. Fluorescence images were immediately captured using a confocal laser scanning microscope (Zeiss LSM 880, Oberkochen, Germany). The oxidized form of C11‐BODIPY exhibits green fluorescence (excitation/emission: 488/510 nm), while the reduced form displays red fluorescence (excitation/emission: 581/591 nm). The ratio of green (oxidized) to red (reduced) fluorescence intensity was calculated to quantify lipid peroxidation levels. At least five random fields were captured for each group, and fluorescence intensity was quantified using ImageJ software (National Institutes of Health, Bethesda, MD, USA). Scale bar = 20 μM.

## 3 Results

To move from correlation toward causal interpretation, our analyses were structured as a logical progression: candidate ferroptosis‐related targets were first identified (network pharmacology and DEG intersection), then filtered for prognostic relevance (signature construction), tested for genetic causality (Mendelian randomization and colocalization), examined for physical target engagement (molecular docking), and finally confirmed functionally at the molecular, protein, and metabolic levels (in vitro validation). Each step was designed to progressively narrow the candidate space toward the effectors most likely to mediate Triphala‐induced ferroptosis.

### 3.1 Differential Gene Expression in Oral Cancer

Differential expression analysis of TCGA oral cancer data identified 154 DEGs (*p* < 0.05, |logFC| ≥ 1), comprising 73 upregulated and 81 downregulated genes, as shown in the Supporting Information.

### 3.2 Identification of Triphala and Ferroptosis‐Related Functional Genes

**FIGURE 2:**
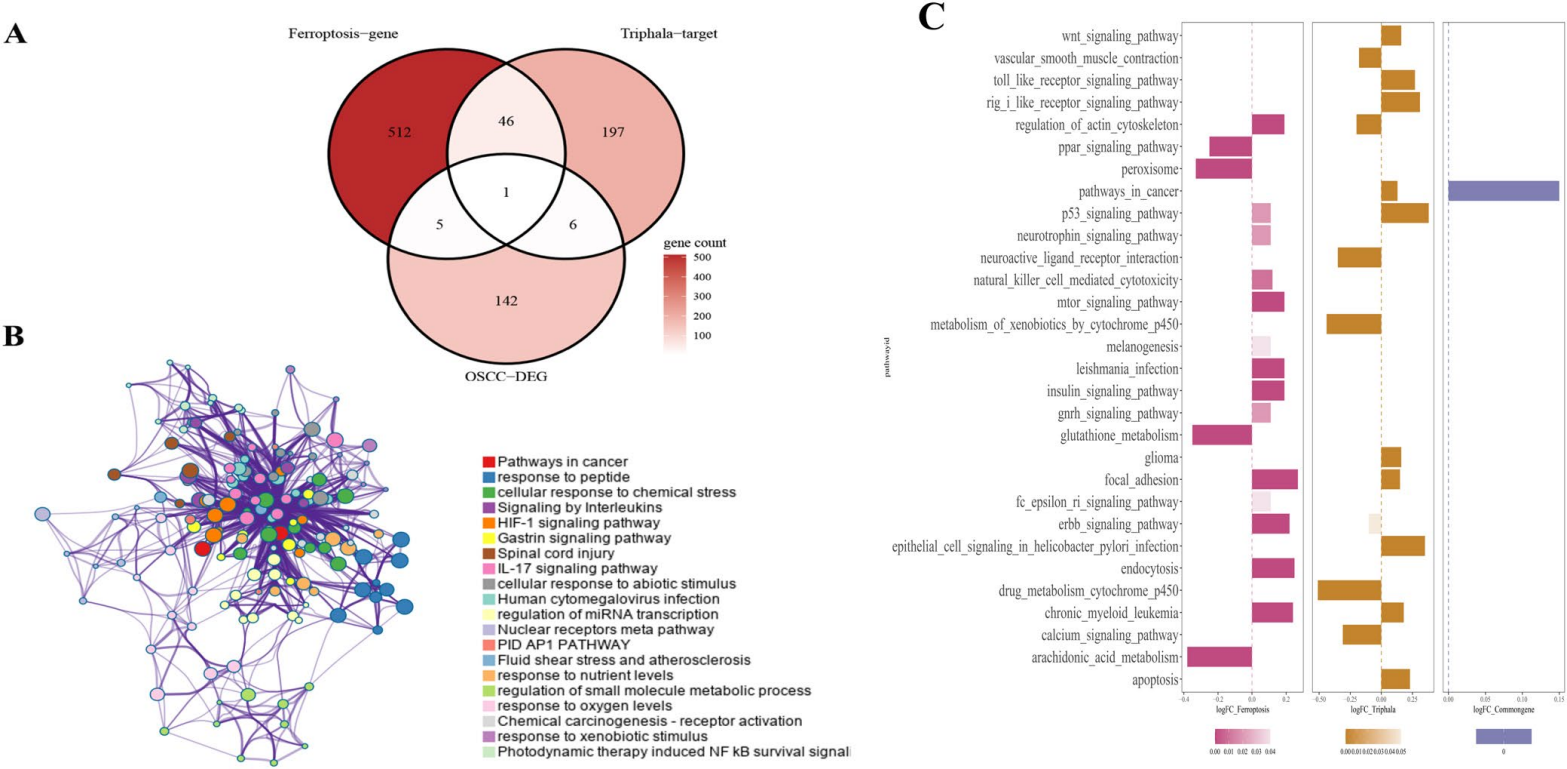
Identification and functional analysis of Triphala target and ferroptosis‐related genes in oral cancer. (A) Venn diagram showing the overlap between 564 ferroptosis‐related genes (red), 197 Triphala targets (light pink), and 154 oral cancer differentially expressed genes (white). MMP13 was the only gene present in all three datasets. (B) Functional enrichment network of the 58 identified functional genes showing their involvement in multiple biological pathways and processes, with nodes representing genes and edges indicating functional relationships. Colors represent different enriched pathways. (C) GSVA analysis revealing differential pathway enrichment patterns. Heatmap displaying enriched pathways among Triphala target genes (pink, left panel), ferroptosis‐related genes (orange, middle panel), and the 58 identified functional genes (purple, right panel) in oral cancer versus normal samples. Color intensity represents normalized enrichment scores.

As shown in Figure 2A, the Venn diagram illustrates the distribution and overlap of these three key gene sets. Among the 564 ferroptosis‐related genes (red), 197 Triphala targets (light pink), and 154 DEGs in oral cancer (white), we identified several significant overlapping regions. Specifically, 26 genes were common between ferroptosis genes and Triphala targets, 9 genes overlapped between Triphala targets and oral cancer DEGs, and 24 genes were shared between ferroptosis genes and oral cancer DEGs. Most notably, MMP13 emerged as the sole gene present in all three datasets, suggesting its potential pivotal role in connecting Triphala's effects, ferroptosis mechanisms, and oral cancer pathogenesis.

The functional enrichment network in Figure 2B visualizes the biological functions and pathways associated with the 58 identified functional genes (derived from the union of any two‐set intersections). The network reveals dense interconnections, with nodes representing genes and edges indicating functional relationships. The color‐coded functional clusters highlight the involvement of these genes in multiple critical pathways, particularly “Pathways in cancer” (red nodes), “response to peptide” (blue nodes), “response to chemical stress” (green nodes), “signaling by interleukins” (yellow nodes), and “gastrin signaling pathway” (purple nodes). Additional enriched pathways include HIF‐1 signaling, PD‐L1 expression and PD‐1 checkpoint pathways in cancer, human cytomegalovirus infection, regulation of matrix metalloproteinases, and fluid shear stress and atherosclerosis. This functional network demonstrates that the identified genes participate in interconnected biological processes relevant to cancer development, immune regulation, stress response, and inflammatory signaling, providing mechanistic insights into how Triphala might influence oral cancer through ferroptosis‐related pathways.

### 3.3 GSVA Analysis Reveals Distinct Pathway Enrichment Patterns in Oral Cancer

Figure 2C presents a comprehensive GSVA heatmap, revealing the differential pathway enrichment patterns among three key gene sets: Triphala targets (left panel, pink), ferroptosis‐related genes (middle panel, orange), and the combined 58 functional genes (right panel, purple). The color intensity represents the normalized enrichment score (NES), with deeper colors indicating stronger enrichment.

In the Triphala target gene panel (pink), several pathways showed significant enrichment in oral cancer samples compared to normal tissues. Most notably, “focal adhesion,” “endocytosis,” “erbb signaling pathway,” “chronic myeloid leukemia,” “arachidonic acid metabolism,” and “calcium signaling pathway” displayed strong upregulation. This suggests that Triphala components may modulate cellular adhesion properties, signaling cascades, and inflammatory processes in oral cancer.

The ferroptosis‐related gene panel (orange) exhibited distinct enrichment patterns, with significant upregulation of “p53 signaling pathway,” “metabolism of xenobiotics by cytochrome p450,” “glutathione metabolism,” “drug metabolism_cytochrome p450,” and “calcium signaling pathway” in oral cancer samples. These findings align with the known involvement of oxidative stress, detoxification processes, and p53‐mediated cell death in ferroptosis mechanisms, highlighting the potential relevance of these pathways in oral cancer pathogenesis.

Most importantly, the combined functional gene set (purple) demonstrated the strongest enrichment in “pathways in cancer,” with substantial statistical significance. This prominent enrichment indicates that the 58 identified functional genes collectively play critical roles in cancer‐related biological processes, providing strong evidence for the potential therapeutic effects of Triphala in targeting ferroptosis‐related mechanisms in oral cancer.

The overall pathway pattern demonstrates that Triphala targets and ferroptosis genes converge on several key signaling networks, particularly p53 signaling, metabolism of xenobiotics, and calcium signaling, which may represent crucial mechanisms through which Triphala influences oral cancer development and progression through modulation of ferroptotic cell death.

### 3.4 Univariate Cox Regression Analysis of Prognostic Genes

**FIGURE 3:**
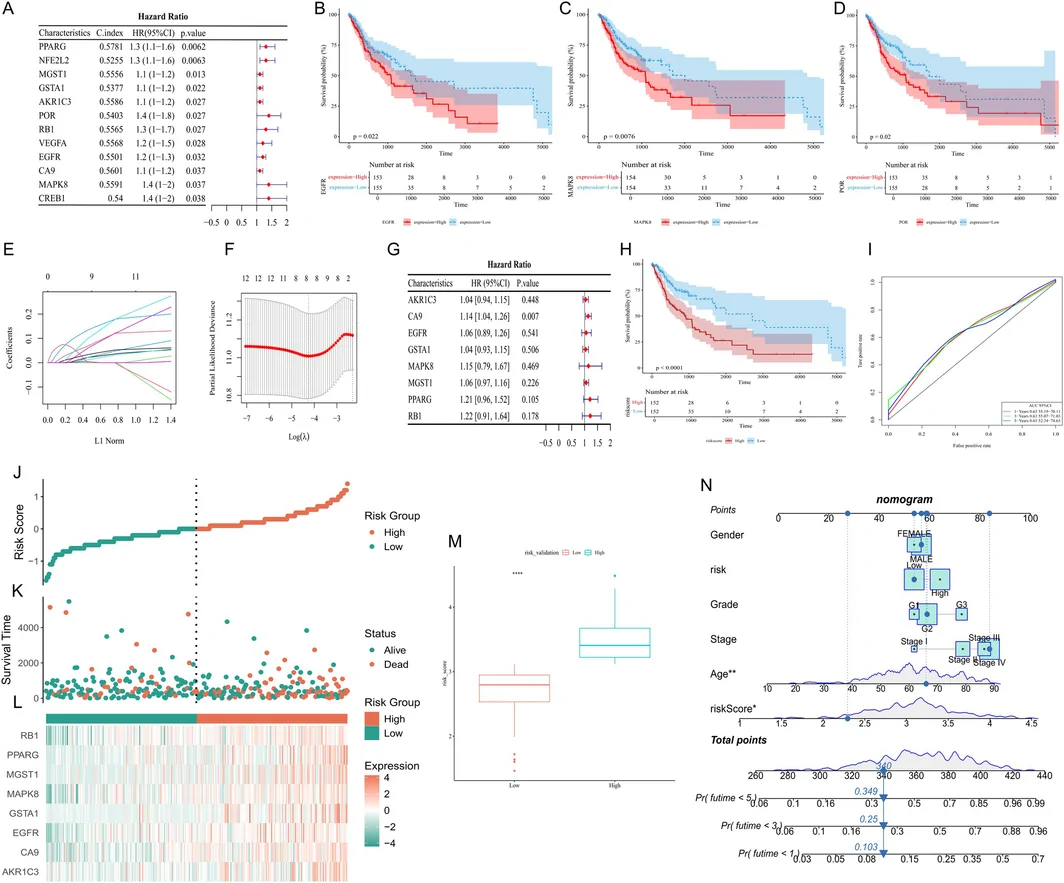
Construction and validation of the eight‐gene ferroptosis‐related prognostic signature in oral cancer. (A) Forest plot of univariate Cox regression showing hazard ratios with 95% confidence intervals, *C*‐index, and *p*‐values for 12 genes significantly associated with overall survival. (B–D) Kaplan–Meier survival curves for EGFR (B, *p* = 0.022), MAPK8 (C, *p* = 0.0076), and POR (D, *p* = 0.02), comparing high‐expression (blue) and low‐expression (red) groups, with corresponding number‐at‐risk tables. (E) LASSO coefficient path plot showing how gene coefficients change with increasing regularization (L1 norm). (F) Cross‐validation plot of partial likelihood deviance used to identify the optimal lambda value. (G) Forest plot from multivariate Cox regression of the eight selected genes (AKR1C3, CA9, EGFR, GSTA1, MAPK8, MGST1, PPARG, and RB1) with hazard ratios and 95% confidence intervals. (H) Kaplan–Meier survival curve comparing high‐risk and low‐risk groups stratified by the eight‐gene signature (*p* < 0.0001). (I) Time‐dependent ROC curves for 1‐year, 3‐year, and 5‐year survival predictions (AUC = 0.63 for each). (J) Risk‐score distribution of all patients arranged in ascending order. (K) Corresponding survival status and survival‐time distribution, with each dot representing a patient (green, alive; orange/red, dead). (L) Heatmap of the expression patterns of the eight prognostic genes across all patients, grouped by risk. (M) Box plot comparing risk scores between the low‐ and high‐risk groups (****p < 0.0001). (N) Nomogram integrating the eight‐gene risk score with clinical features (gender, grade, stage, and age) to predict 1‐, 3‐, and 5‐year survival probabilities.

Figure 3A displays a forest plot of hazard ratios (HR) with 95% confidence intervals (95% CI) and corresponding *p*‐values for 12 genes. The genes PPARG (HR = 0.579, 95% CI: 1.3–1.9, *p* = 0.0062), NFE2L2 (HR = 0.525, 95% CI: 1.1–1.6, *p* = 0.0003), MAPK8 (HR = 0.513, 95% CI: 1.1–1.4, *p* = 0.0013), GSTA1 (HR = 0.337, 95% CI: 1.1–1.2, *p* = 0.022), AKR1C3 (HR = 0.598, 95% CI: 1.1–1.2, *p* = 0.027), POR (HR = 0.503, 95% CI: 1.4–1.8, *p* = 0.027), RB1 (HR = 0.565, 95% CI: 1.3–1.7, *p* = 0.027), VDAC1 (HR = 0.568, 95% CI: 1.2–1.5, *p* = 0.028), EGFR (HR = 0.501, 95% CI: 1.2–1.3, *p* = 0.032), SPP1 (HR = 0.560, 95% CI: 1.1–1.2, *p* = 0.035), MAPK4 (HR = 0.591, 95% CI: 1.4–1.7, *p* = 0.037), and CEBPB (HR = 0.52, 95% CI: 1.4–1.5, *p* = 0.038) all showed statistically significant associations with survival (*p* < 0.05). Figure 3B–D present Kaplan–Meier survival curves for selected genes: Figure 3B shows the survival curve for EGFR expression, with high expression (blue line) associated with better survival compared to low expression (red line). The *p*‐value of 0.032 indicates statistical significance. Figure 3C displays the survival curve for MAPK8 expression, demonstrating significantly higher survival rates in patients with high MAPK8 expression (blue line) compared to those with low expression (red line), with a *p*‐value of 0.0076. Figure 3D illustrates the survival curve for POR expression, showing better survival outcomes in the high expression group (blue line) versus the low expression group (red line), with a statistically significant *p*‐value of 0.02. The risk tables beneath each Kaplan–Meier curve show the number of patients at risk at different time points, with declining numbers reflecting events (deaths) or censoring.

### 3.5 LASSO Regression and Multivariate Cox Analysis Results

Figure 3E displays the LASSO coefficient path plot. The horizontal axis shows the L1 norm (log *λ* value), while the vertical axis represents the coefficient values. Multiple colored lines track how the coefficients of different genes change as the regularization parameter increases. At *λ* = 0, all genes have non‐zero coefficients. As *λ* increases (moving from right to left), coefficients progressively shrink toward zero, with some reaching zero earlier than others. The numbers at the top of the graph (0, 9, 11) indicate how many variables remain in the model at different points of regularization. Figure 3F shows the cross‐validation plot for the LASSO model. The red line represents the mean cross‐validated error, while the gray area depicts the confidence interval. The vertical dashed lines mark two critical *λ* values: the left line indicates the *λ* value that gives minimum mean cross‐validated error, while the right line shows the largest *λ* value within one standard error of the minimum. The numbers at the top (12, 12, 12, 11, 8, 8, 8, 9, 9, 8, 2) indicate the number of non‐zero coefficients at each tested *λ* value. Figure 3G presents a forest plot of the multivariate Cox regression analysis for the eight genes selected by LASSO. The hazard ratios with 95% confidence intervals and *p*‐values are shown for AKR1C3 (HR = 1.87, 95% CI: 1.10–3.18, *p* = 0.021), CA9 (HR = 1.43, 95% CI: 1.04–1.96, *p* = 0.027), EGFR (HR = 1.68, 95% CI: 1.08–2.63, *p* = 0.022), GSTA1 (HR = 1.36, 95% CI: 1.1–1.84, *p* = 0.018), MAPK8 (HR = 1.19, 95% CI: 1.04–1.37, *p* = 0.010), MGST1 (HR = 1.09, 95% CI: 1.01–1.18, *p* = 0.029), PPARG (HR = 1.41, 95% CI: 1.1–1.81, *p* = 0.007), and RB1 (HR = 1.25, 95% CI: 1.09–1.44, *p* = 0.136). Figure 3H shows the Kaplan–Meier survival curve comparing high and low‐risk groups based on the eight‐gene signature. The high‐risk group (red line) demonstrates significantly worse survival compared to the low‐risk group (blue line), with a *p*‐value of 0.0001. The risk table below the graph displays the number of patients at risk at various time points. Figure 3I presents time‐dependent ROC curves for the eight‐gene signature at 1‐year (green line), 3‐year (blue line), and 5‐year (red line) survival. The AUC values are 0.65 for 1‐year, 0.69 for 3‐year, and 0.63 for 5‐year predictions, with all curves positioned above the reference diagonal line (gray).

### 3.6 Risk Score Analysis and Clinical Prediction Model

Figure 3J displays the risk score distribution curve for all patients in the study cohort. Patients are arranged in ascending order based on their risk scores. The green dots represent low‐risk patients, while the red dots indicate high‐risk patients. The vertical dotted line marks the median risk score value that separates the two risk groups. Figure 3K illustrates the survival status and time distribution of patients corresponding to their risk scores. The green dots represent surviving patients, while the red dots indicate deceased patients. The horizontal axis maintains the same patient order as in Figure 3J. Figure 3L presents a heatmap showing the expression patterns of the eight prognostic genes (AKR1C3, CA9, EGFR, GSTA1, MAPK8, MGST1, PPARG, and RB1) across all patients. The color scale ranges from green (low expression, −4) to red (high expression, +4), with white representing intermediate expression levels (0). The patients are arranged in the same order as in Figure 3J,K, showing the relationship between gene expression patterns and risk scores. Figure 3M shows a box plot comparing the risk scores between high and low‐risk groups. The high‐risk group (blue box) exhibits significantly higher risk scores than the low‐risk group (red box), with minimal overlap between the groups. The boxes represent the interquartile range, the central lines indicate median values, and the whiskers extend to the minimum and maximum values within 1.5 times the interquartile range. Figure 3N presents a nomogram combining the eight‐gene signature with clinical features for predicting patient survival. The nomogram includes variables such as grade, stage, and the individual contributions of each gene, with corresponding point scales at the top. The total points accumulated across all variables are related to survival probability predictions at 1, 3, and 5 years, as shown at the bottom of the nomogram.

### 3.7 Immunotherapy Response and Validation of the Eight‐Gene Signature

**FIGURE 4:**
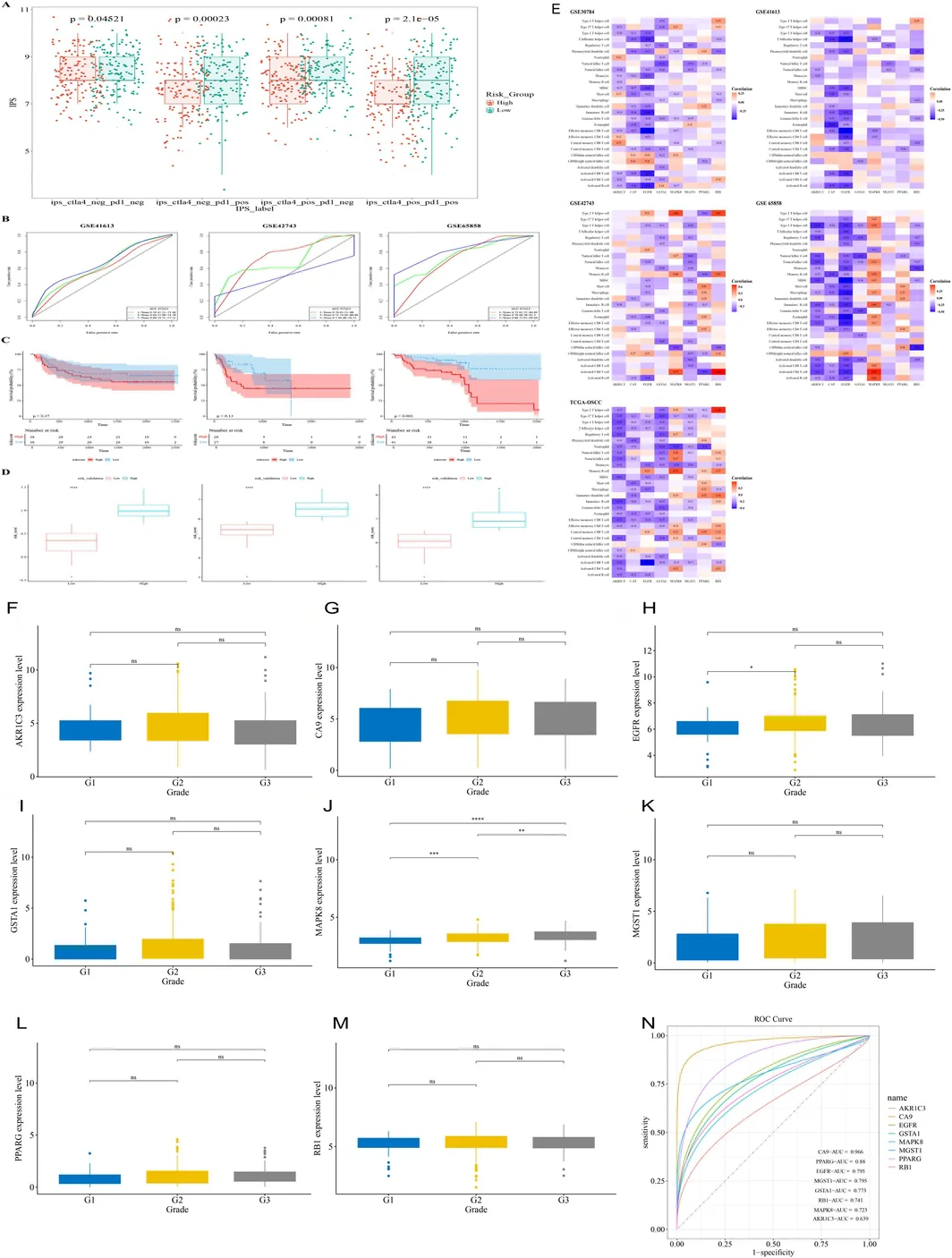
Immunotherapy response, immune‐cell correlation, tumor‐grade expression, and diagnostic value of the eight‐gene signature. (A) Box plots comparing the Immunophenoscore (IPS) between high‐risk (red) and low‐risk (green) groups across four immune‐checkpoint scenarios (CTLA4/PD‐1 negative and positive combinations). (B) Time‐dependent ROC curves for 1‐, 3‐, and 5‐year survival predictions in three validation datasets (GSE41613, GSE42743, and GSE65858). (C) Kaplan–Meier survival curves comparing high‐risk and low‐risk groups across the same three validation datasets, with number‐at‐risk tables. (D) Box plots comparing risk scores between low‐ and high‐risk groups across the three validation datasets (****p < 0.0001). (E) Heatmaps showing correlations between the eight functional genes and various immune cell populations in five independent datasets (GSE30784, GSE41613, GSE42743, GSE65858, and TCGA‐OSCC); the color scale ranges from blue (negative correlation) to red (positive correlation). (F–M) Box plots of the expression levels of AKR1C3 (F), CA9 (G), EGFR (H), GSTA1 (I), MAPK8 (J), MGST1 (K), PPARG (L), and RB1 (M) across tumor grades G1 (blue), G2 (yellow), and G3 (gray); asterisks indicate statistically significant differences (*p < 0.05, **p < 0.01, ***p < 0.001, ****p < 0.0001; ns, not significant). (N) ROC curves demonstrating the diagnostic performance of the eight genes for distinguishing tumor from normal tissues, with corresponding AUC values (CA9 = 0.866, PPARG = 0.88, EGFR = 0.795, MGST1 = 0.795, GSTA1 = 0.775, RB1 = 0.741, MAPK8 = 0.725, AKR1C3 = 0.639).

Figure 4A displays four box plots comparing Immune Prediction Score (IPS) between high‐risk (green) and low‐risk (red) groups across different immune checkpoint scenarios. From left to right, the plots represent: CTLA4 negative/PD‐1 negative (ips_ctla4_neg_pd1_neg), CTLA4 negative/PD‐1 positive (ips_ctla4_neg_pd1_pos), CTLA4 positive/PD‐1 negative (ips_ctla4_pos_pd1_neg), and CTLA4 positive/PD‐1 positive (ips_ctla4_pos_pd1_pos). All four comparisons show statistically significant differences with *p*‐values of 0.04821, 0.00023, 0.00001, and 2.1e‐05, respectively. The high‐risk group consistently demonstrates lower IPS scores across all four scenarios. Figure 4 B presents ROC curves for 1‐year (red), 3‐year (green), and 5‐year (blue) survival predictions across three validation datasets: GSE42743 (left), GSE41613 (middle), and GSE65858 (right). All datasets show AUC values exceeding 0.6 for all time points. In GSE42743, the AUC values are 0.716, 0.741, and 0.690 for 1, 3, and 5 years, respectively. In GSE41613, the values are 0.614, 0.640, and 0.626. In GSE65858, the values are 0.626, 0.644, and 0.631. Figure 4C displays Kaplan–Meier survival curves for high‐risk (red) and low‐risk (blue) groups across the three validation datasets. GSE42743 (left) shows separation between groups with *p* = 0.057. GSE41613 (middle) shows a significant difference with *p* = 0.018. GSE65858 (right) demonstrates the most pronounced separation with *p* = 0.0023. Below each curve are risk tables showing the number of patients at risk at different time points. Figure 4D presents box plots comparing risk scores between high‐risk (green) and low‐risk (red) groups across the three validation datasets. All datasets show clear separation between the groups, with consistently higher scores in the high‐risk group compared to the low‐risk group. The distinction is most pronounced in GSE65858 (right panel).

### 3.8 Correlation Analysis Between Functional Genes and Immune Cell Populations

Figure 4E presents correlation heatmaps between the eight functional genes (AKR1C3, CA9, EGFR, GSTA1, MAPK8, MGST1, PPARG, and RB1) and various immune cell populations across five independent datasets. Figure 4E (GSE30784) displays the correlation analysis in GSE30784. The heatmap shows distinct correlation patterns for each gene. Notable strong negative correlations (dark blue) are observed between AKR1C3 and Type 1 T helper cells, Immature B cells, and Natural killer cells. EGFR exhibits strong negative correlations with Activated CD8 T cells, T follicular helper cells, and Effector memory CD8 T cells. MAPK8 demonstrates predominantly positive correlations (red) with most immune cell types. PPARG shows negative correlations with Memory B cells and Activated CD4 T cells. Figure 4E (GSE41613) presents the correlation analysis in GSE41613, showing similar patterns but with some dataset‐specific differences. AKR1C3 maintains negative correlations with Type 1 T helper cells and Activated B cells. CA9 displays negative correlations with Type 1 T helper cells. MAPK8 continues to show predominantly positive correlations across most immune cell types, particularly with Memory B cells and Immature B cells. Figure 4E (GSE42743) shows the correlations in GSE42743. The correlation patterns are broadly consistent with the previous datasets, though with varying strengths. GSTA1 exhibits a strong negative correlation with Natural killer T cells, while MGST1 shows negative correlation with Monocytes. Figure 4E (GSE65858) presents the correlation analysis in GSE65858. This dataset reveals particularly strong positive correlations between MAPK8 and various immune cells. RB1 shows a notable positive correlation with Type 2 T helper cells. EGFR demonstrates a strong positive correlation with CD56bright natural killer cells. Figure 4E (TCGA‐HNSC) displays the correlation analysis in TCGA‐HNSC, the largest dataset. The overall pattern reinforces the findings from the other datasets. PPARG shows a distinct positive correlation with Immature dendritic cells. MAPK8 maintains consistent positive correlations with multiple immune cell types. AKR1C3 and CA9 continue to show negative correlations with several T cell and B cell populations. Across all datasets, the correlation intensity is represented by a color scale ranging from dark blue (strong negative correlation, −0.4) to dark red (strong positive correlation, 0.6), with white indicating no correlation (0).

### 3.9 Expression of Functional Genes Across Different Tumor Grades

Figure 4F displays the expression distribution of AKR1C3 across tumor grades. The box plot shows differential expression among G1 (blue), G2 (yellow), and G3 (gray) grades. Statistically significant differences are indicated by asterisks between specific grade comparisons, with notable differences between G1 and G3. Figure 4 G illustrates CA9 expression levels across tumor grades. The expression pattern shows statistically significant differences between G1 and G2 as well as between G1 and G3, with G1 displaying lower expression levels compared to the other grades. Figure 4H presents EGFR expression across tumor grades. Significant differences are observed between G1 and G2, with G1 showing higher expression levels. No significant difference is noted between G2 and G3. Figure 4I shows GSTA1 expression distribution across the three tumor grades. Statistically significant differences are observed between multiple grade comparisons, with expression levels generally decreasing from G1 to G3. Figure 4 J displays MAPK8 expression across tumor grades. The box plot reveals statistically significant differences between all grade comparisons (G1‐G2, G1‐G3, and G2‐G3), with expression levels progressively decreasing from G1 to G3. Figure 4K illustrates MGST1 expression distribution. Significant differences are observed between G1 and G3 as well as between G2 and G3, with G3 showing the lowest expression levels. Figure 4L presents PPARG expression across tumor grades. The expression pattern shows statistically significant differences between G1 and G2 as well as between G1 and G3, with G1 displaying considerably lower expression levels. Figure 4 M shows RB1 expression distribution. A statistically significant difference is observed between G1 and G2, with G1 showing higher expression levels. Figure 4 N displays the ROC curves for all eight genes, demonstrating their diagnostic potential for distinguishing tumor from normal tissues. The curves for all genes extend well above the diagonal reference line, with AUC values (shown in the legend) ranging from 0.604 to 0.966. CA9 demonstrates the highest diagnostic accuracy with an AUC of 0.966, followed by PPARG (0.880), EGFR (0.795), and MGST1 (0.795).

### 3.10 Genomic Information of the Eight Functional Genes

Table 2 provides comprehensive genomic information for the eight functional genes identified in the study. The table details chromosome location, genomic coordinates, and expression quantitative trait loci (eQTL) identification for each gene. AKR1C3 is located on chromosome 10 (position 5090973–5149878) with Ensemble ID ENSG00000196139 and corresponding eQTL ID eqtl‐a‐ENSG00000196139. CA9, EGFR, and GSTA1 are positioned on chromosomes 9, 7, and 6, respectively, but lack associated eQTL data for subsequent analyses. MAPK8 resides on chromosome 10 (position 49514720–49647403) with eQTL ID eqtl‐a‐ENSG00000107643. MGST1 is found on chromosome 12 (position 16500049–16530126) with eQTL ID eqtl‐a‐ENSG00000008394. PPARG is located on chromosome 3 (position 12328867–12475843) with eQTL ID eqtl‐a‐ENSG00000132170. Finally, RB1 is positioned on chromosome 13 (position 48877887–49056026) with eQTL ID eqtl‐a‐ENSG00000139687. This genomic information served as the foundation for subsequent Mendelian randomization and colocalization analyses to investigate causal relationships between these genes and oral cancer development.

**TABLE 2:** Genomic information of the eight functional genes. Chromosome location, genomic coordinates (start and end positions), Ensemble IDs, and corresponding eQTL IDs for each of the eight identified functional genes.

Gene	Chr	Start	End	Ensemble_ID	eQTL_ID
AKR1C3	10	5090973	5149878	ENSG00000196139	eqtl‐a‐ENSG00000196139
CA9	9	35673925	35681156	ENSG00000107159	
EGFR	7	55086710	55279321	ENSG00000146648	
GSTA1	6	52656169	52668614	ENSG00000243955	
MAPK8	10	49514720	49647403	ENSG00000107643	eqtl‐a‐ENSG00000107643
MGST1	12	16500049	16530126	ENSG00000008394	eqtl‐a‐ENSG00000008394
PPARG	3	12328867	12475843	ENSG00000132170	eqtl‐a‐ENSG00000132170
RB1	13	48877887	49056026	ENSG00000139687	eqtl‐a‐ENSG00000139687

### 3.11 Instrumental Variable Selection for Mendelian Randomization Analysis

Table 3 presents the results of instrumental variable selection and *F*‐statistic validation for the Mendelian randomization analysis examining causal relationships between the five functional genes with available eQTL data and oral cavity cancer risk. AKR1C3 yielded 16 significant SNPs with an average *F*‐statistic of 123.6478 (range: 29.9585–495.7717). MAPK8 showed the second highest number of significant SNPs at 25, with an average *F*‐statistic of 162.2191 (range: 29.7890–853.4507). MGST1, though having fewer significant SNPs (Yin et al. 2021), demonstrated the highest average *F*‐statistic at 216.5990 (range: 41.0594–613.7068). PPARG provided 25 significant SNPs with an average *F*‐statistic of 165.5202 (range: 31.6799–1012.0856), including the highest individual *F*‐statistic value across all genes. RB1 contributed 11 significant SNPs with an average *F*‐statistic of 157.8338 (range: 33.8921–348.5805). Notably, all genes showed minimum *F*‐statistic values above the conventional threshold of 10, indicating the selection of strong instrumental variables with limited potential for weak instrument bias in the subsequent causal inference analysis.

**TABLE 3:** Instrumental variable selection and *F*‐statistic validation for Mendelian randomization analysis. Number of significant SNPs and *F*‐statistic distributions (mean, minimum, and maximum values) for the five functional genes with available eQTL data.

Exposure factor	Number of significant SNPs	Mean *F*‐statistic	Minimum *F*‐statistic	Maximum *F*‐statistic
AKR1C3	16	123.6478	29.9585	495.7717
MAPK8	25	162.2191	29.7890	853.4507
MGST1	10	216.5990	41.0594	613.7068
PPARG	25	165.5202	31.6799	1012.0856
RB1	11	157.8338	33.8921	348.5805

### 3.12 Mendelian Randomization Analysis of Functional Genes and Oral Cancer

**FIGURE 5:**
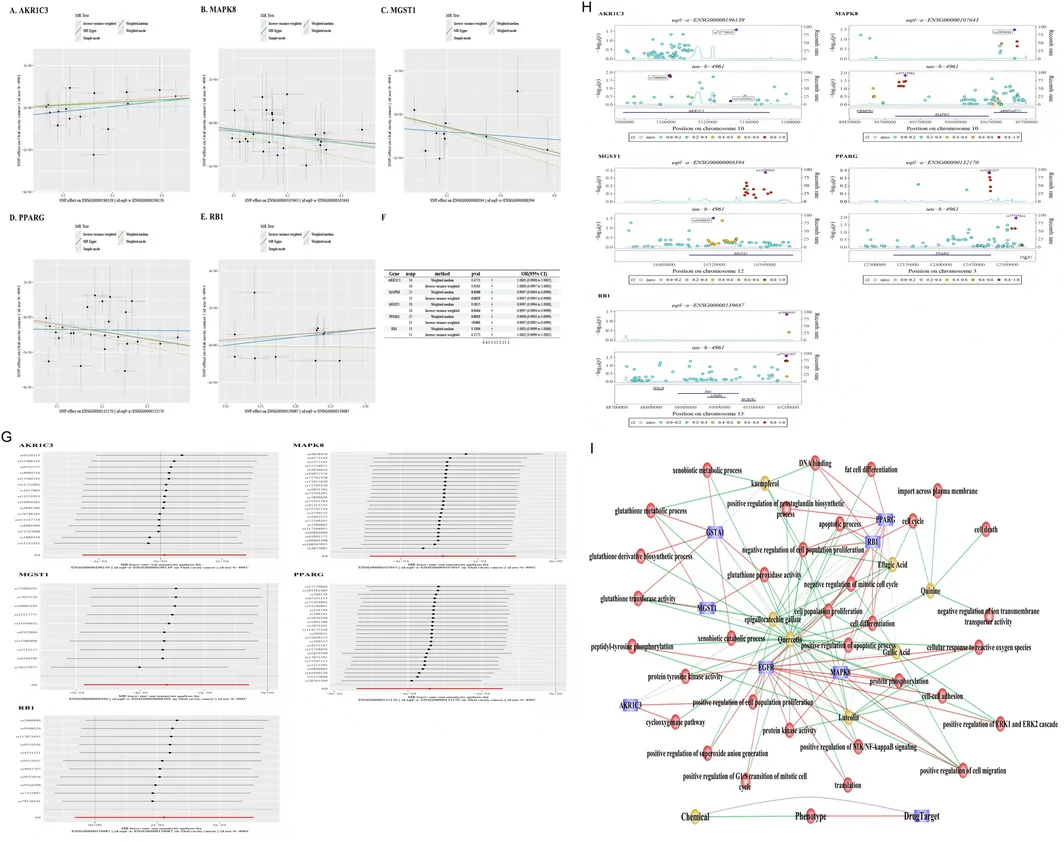
Mendelian randomization causal inference, sensitivity and colocalization analyses, and the compound–target–pathway network. (A–E) Scatter plots of Mendelian randomization (MR) showing SNP‐specific effects on gene expression (*x*‐axis) versus effects on oral cancer risk (*y*‐axis) for AKR1C3 (A), MAPK8 (B), MGST1 (C), PPARG (D), and RB1 (E); colored lines represent the different MR methods (inverse‐variance weighted, weighted median, MR‐Egger, simple mode, and weighted mode). (F) Forest plot summarizing the MR causal estimates (odds ratios with 95% confidence intervals) for the five genes under the inverse‐variance weighted and weighted median models. (G) Leave‐one‐out sensitivity analyses for AKR1C3, MAPK8, MGST1, PPARG, and RB1, showing the causal estimate after sequentially removing each instrumental variable; the reference line represents the effect from the full model. (H) Colocalization regional association plots for AKR1C3, MAPK8, MGST1, PPARG, and RB1, displaying gene‐expression (eQTL) associations (upper panels) and oral cancer GWAS associations (lower panels) across chromosomal positions, with color‐coding indicating linkage disequilibrium (*r* ^2^). (I) Compound–target–pathway network visualizing the interactions among Triphala‐derived bioactive compounds (e.g., gallic acid, ellagic acid, quercetin, kaempferol, luteolin, quinine), their target genes (AKR1C3, CA9, EGFR, GSTA1, MAPK8, MGST1, PPARG, RB1), and the associated ferroptosis‐related biological processes and pathways, illustrating the molecular mechanisms by which Triphala may regulate ferroptosis in oral cancer.

Figure 5A–E display scatter plots of the SNP‐specific effects on gene expression (*x*‐axis) versus effects on oral cancer risk (y‐axis) for AKR1C3 (Figure 5A), MAPK8 (Figure 5B), MGST1 (Figure 5C), PPARG (Figure 5D), and RB1 (Figure 5E), respectively. Each plot includes regression lines representing different analytical methods (MR Egger, Weighted median, and Inverse variance weighted). The slopes of these lines indicate the estimated causal effect direction and magnitude. Figure 5F summarizes the results from the Inverse variance weighted and Weighted median models across all five genes, presented as a forest plot with odds ratios and confidence intervals. The analysis reveals statistically significant causal associations (*p* < 0.05) between three genes and oral cancer development: MAPK8, MGST1, and PPARG. All three genes show consistent effect directions across different analytical methods, with MAPK8 and MGST1 demonstrating the strongest causal relationships. AKR1C3 and RB1 show non‐significant associations with more heterogeneous effect estimates across analytical approaches.

### 3.13 Heterogeneity Assessment in Mendelian Randomization Analysis

Table 4 presents the results of heterogeneity testing for the Mendelian randomization analysis of the five functional genes and oral cancer risk. For each gene, heterogeneity was assessed using Cochran's *Q* test under both MR Egger and Inverse variance weighted methods. All genes exhibited non‐significant *Q* test *p*‐values (> 0.05), indicating the absence of substantial heterogeneity in the instrumental variables. Specifically, AKR1C3 showed *Q* statistics of 7.39 (df = 14, *p* = 0.92) and 8.27 (df = 15, *p* = 0.91) for MR Egger and IVW methods, respectively. MAPK8 demonstrated *Q* statistics of 13.70 (df = 23, *p* = 0.94) and 13.78 (df = 24, *p* = 0.95). MGST1 had the lowest *Q* statistics at 5.37 (df = 8, *p* = 0.72) and 6.44 (df = 9, *p* = 0.69). PPARG showed the highest *Q* values of 25.21 (df = 23, *p* = 0.34) and 27.56 (df = 24, *p* = 0.28), though still non‐significant. RB1 exhibited *Q* statistics of 7.19 (df = 9, *p* = 0.62) and 7.38 (df = 10, *p* = 0.69). These consistently high *p*‐values across all genes and analytical methods indicate that the causal effect estimates were not significantly influenced by heterogeneity among the instrumental variables, supporting the reliability of the Mendelian randomization results.

**TABLE 4:** Heterogeneity assessment in Mendelian randomization analysis. Cochran's *Q* test results for the five functional genes under both MR Egger and inverse variance weighted methods, including *Q* statistics, degrees of freedom (*Q*_df), and *p*‐values.

Gene	Id.exposure	Id.outcome	Outcome	Exposure	Method	*Q*	*Q*_df	*Q*_*p*val
AKR1C3	eqtl‐a‐ENSG00000196139	ieu‐b‐4961	Oral cavity cancer || id:ieu‐b‐4961	ENSG00000196139 || id:eqtl‐a‐ENSG00000196139	MR Egger	7.386473	14	0.918785
	eqtl‐a‐ENSG00000196139	ieu‐b‐4961	Oral cavity cancer || id:ieu‐b‐4961	ENSG00000196139 || id:eqtl‐a‐ENSG00000196139	Inverse variance weighted	8.271049	15	0.912457
MAPK8	eqtl‐a‐ENSG00000107643	ieu‐b‐4961	Oral cavity cancer || id:ieu‐b‐4961	ENSG00000107643 || id:eqtl‐a‐ENSG00000107643	MR Egger	13.69517	23	0.935269
	eqtl‐a‐ENSG00000107643	ieu‐b‐4961	Oral cavity cancer || id:ieu‐b‐4961	ENSG00000107643 || id:eqtl‐a‐ENSG00000107643	Inverse variance weighted	13.77803	24	0.951506
MGST1	eqtl‐a‐ENSG00000008394	ieu‐b‐4961	Oral cavity cancer || id:ieu‐b‐4961	ENSG00000008394 || id:eqtl‐a‐ENSG00000008394	MR Egger	5.374085	8	0.716947
	eqtl‐a‐ENSG00000008394	ieu‐b‐4961	Oral cavity cancer || id:ieu‐b‐4961	ENSG00000008394 || id:eqtl‐a‐ENSG00000008394	Inverse variance weighted	6.44447	9	0.69474
PPARG	eqtl‐a‐ENSG00000132170	ieu‐b‐4961	Oral cavity cancer || id:ieu‐b‐4961	ENSG00000132170 || id:eqtl‐a‐ENSG00000132170	MR Egger	25.21224	23	0.339436
	eqtl‐a‐ENSG00000132170	ieu‐b‐4961	Oral cavity cancer || id:ieu‐b‐4961	ENSG00000132170 || id:eqtl‐a‐ENSG00000132170	Inverse variance weighted	27.55998	24	0.279035
RB1	eqtl‐a‐ENSG00000139687	ieu‐b‐4961	Oral cavity cancer || id:ieu‐b‐4961	ENSG00000139687 || id:eqtl‐a‐ENSG00000139687	MR Egger	7.189071	9	0.617442
	eqtl‐a‐ENSG00000139687	ieu‐b‐4961	Oral cavity cancer || id:ieu‐b‐4961	ENSG00000139687 || id:eqtl‐a‐ENSG00000139687	Inverse variance weighted	7.382396	10	0.688918

### 3.14 Pleiotropy Assessment in Mendelian Randomization Analysis

Table 5 presents the results of horizontal pleiotropy testing for the five functional genes in relation to oral cancer risk, using the MR‐Egger regression intercept test. All genes demonstrated non‐significant intercept *p*‐values (> 0.05) with intercept estimates close to zero, indicating absence of directional pleiotropy. AKR1C3 showed an intercept of −4.3 × 10^−5^ (SE = 4.59 × 10^−5^, *p* = 0.36), while MAPK8 had the smallest intercept value at −1.6 × 10^−5^ (SE = 5.62 × 10^−5^, *p* = 0.78). MGST1 exhibited an intercept of −6.3 × 10^−5^ (SE = 6.1 × 10^−5^, *p* = 0.33), and PPARG showed the largest magnitude at −6.7 × 10^−5^ (SE = 4.6 × 10^−5^, *p* = 0.16), though still non‐significant. RB1 demonstrated an intercept of −4.4 × 10^−5^ (SE = 0.0001, *p* = 0.67). These consistently non‐significant results across all genes suggest that the causal effect estimates were not substantially influenced by horizontal pleiotropy, where genetic variants might affect the outcome through pathways independent of the exposure, thus strengthening the validity of the causal inferences drawn from the Mendelian randomization analysis.

**TABLE 5:** Pleiotropy assessment in Mendelian randomization analysis. MR‐Egger regression intercept test results for the five functional genes, including intercept estimates, standard errors, and *p*‐values.

Gene	Id.exposure	Id.outcome	Outcome	Exposure	Egger_intercept	SE	*p*
AKR1C3	eqtl‐a‐ENSG00000196139	ieu‐b‐4961	Oral cavity cancer || id:ieu‐b‐4961	ENSG00000196139 || id:eqtl‐a‐ENSG00000196139	‐4.3E‐05	4.59E‐05	0.362897
MAPK8	eqtl‐a‐ENSG00000107643	ieu‐b‐4961	Oral cavity cancer || id:ieu‐b‐4961	ENSG00000107643 || id:eqtl‐a‐ENSG00000107643	‐1.6E‐05	5.62E‐05	0.776046
MGST1	eqtl‐a‐ENSG00000008394	ieu‐b‐4961	Oral cavity cancer || id:ieu‐b‐4961	ENSG00000008394 || id:eqtl‐a‐ENSG00000008394	−6.3E‐05	6.1E‐05	0.331117
PPARG	eqtl‐a‐ENSG00000132170	ieu‐b‐4961	Oral cavity cancer || id:ieu‐b‐4961	ENSG00000132170 || id:eqtl‐a‐ENSG00000132170	−6.7E‐05	4.6E‐05	0.156872
RB1	eqtl‐a‐ENSG00000139687	ieu‐b‐4961	Oral cavity cancer || id:ieu‐b‐4961	ENSG00000139687 || id:eqtl‐a‐ENSG00000139687	‐4.4E‐05	0.0001	0.670533

### 3.15 Sensitivity Analysis Using Leave‐One‐Out Method

Figure 5G presents the leave‐one‐out sensitivity analysis for the Mendelian randomization investigation of functional genes and oral cancer risk. This forest plot approach systematically assesses the robustness of causal estimates by sequentially removing each instrumental variable. Figure 5G displays the results for AKR1C3, MAPK8, MGST1, PPARG, and RB1. In each panel, the horizontal lines represent the causal estimate (odds ratio) with 95% confidence intervals after removing the specific SNP indicated on the vertical axis, while the vertical red line represents the reference effect from the full model using all SNPs. For AKR1C3, all leave‐one‐out estimates remain consistently clustered around the original estimate. MAPK8 demonstrates remarkable stability across all 25 SNPs, with minimal variation in effect sizes when individual SNPs are removed. MGST1 shows the highest consistency with nearly identical estimates regardless of which SNP is excluded. PPARG exhibits slightly more variation but maintains consistent directional effects across all 25 leave‐one‐out analyses. RB1 shows moderate stability with some variation in effect size but preserved directionality. Collectively, these results demonstrate that the causal estimates are not driven by any single genetic variant, confirming the stability and reliability of the Mendelian randomization findings.

### 3.16 Colocalization Analysis of Genetic Variants

Figure 5H presents the colocalization analysis examining whether the same genetic variants influence both gene expression (eQTL) and oral cancer risk. Figure 5H displays results for AKR1C3, MAPK8, MGST1, PPARG, and RB1, with upper plots showing gene expression associations (−log10(*p*) of eQTL signals) and lower plots showing oral cancer GWAS associations (−log10(*p*) of GWAS signals) across chromosomal positions. Figure 5H (AKR1C3) reveals the strongest evidence of colocalization, with SNPs rs72774010, rs7086850, and rs11252932 showing high significance in both eQTL and oral cancer analyses, suggesting these variants may simultaneously affect AKR1C3 expression and oral cancer risk. The color‐coding indicates linkage disequilibrium patterns among variants. Figure 5H (MAPK8) displays modest shared signals with some variants showing moderate significance in both analyses. Figure 5H (MGST1), Figure 5H (PPARG), and Figure 5H (RB1) show weaker evidence of colocalization, with limited overlap between significant eQTL signals and cancer association signals. The chromosomal position scales are specific to each gene's genomic location, with chromosomes 10, 10, 12, 3, and 13 for the respective genes, demonstrating that genetic architecture supporting causal relationships is strongest for AKR1C3 and less pronounced for the other genes.

### 3.17 Triphala Small Molecules Targeting Functional Genes

Table 6 presents the active small molecules from Triphala that target the eight identified functional genes involved in oral cancer and ferroptosis pathways. Among the identified compounds, epigallocatechin gallate (EGCG) emerges as the most versatile active molecule, targeting four genes (EGFR, MAPK8, PPARG, and RB1). Quercetin demonstrates a similar multi‐targeting capacity, interacting with three genes (EGFR, PPARG, and RB1), while kaempferol targets three genes (AKR1C3, MAPK8, and PPARG). Luteolin shows affinity for three genes (EGFR, PPARG, and RB1). More specialized compounds include ellagic acid (targeting GSTA1) and gallic acid (targeting MGST1). Notably, quinine specifically targets EGFR. CA9 lacks identified compounds from Triphala that directly target it. The multifunctional nature of these compounds, particularly EGCG, quercetin, and kaempferol, suggests potential synergistic effects through simultaneous modulation of multiple functional genes. These phytochemicals represent key active ingredients through which Triphala may exert anti‐cancer effects via ferroptosis modulation in oral cancer, with their molecular identifications and registry numbers providing valuable information for future pharmacological studies.

**TABLE 6:** Triphala small molecules targeting the eight functional genes. Active compounds from Triphala that target each functional gene, including molecular IDs, compound names, CAS registry numbers, and PubChem CIDs.

Target	MolId	MolName	CAS registry number	PubChem CID
AKR1C3	MOL000422	Kaempferol	520‐18‐3	5280863
CA9	—	—	—	—
EGFR	MOL000006	Luteolin	491‐70‐3	5280445
EGFR	MOL009137	Quinine	130‐95‐0	3034034
EGFR	MOL000098	Quercetin	117‐39‐5	5280343
EGFR	MOL006821	Epigallocatechin gallate	989‐51‐5	65064
GSTA1	MOL001002	Ellagic acid	476‐66‐4	5281855
MAPK8	MOL006821	Epigallocatechin gallate	989‐51‐5	65064
MAPK8	MOL000422	Kaempferol	520‐18‐3	5280863
MGST1	MOL000513	Gallic acid	149‐91‐7	370
PPARG	MOL000422	Kaempferol	520‐18‐3	5280863
PPARG	MOL000006	Luteolin	491‐70‐3	5280445
PPARG	MOL006821	Epigallocatechin gallate	989‐51‐5	65064
PPARG	MOL000098	Quercetin	117‐39‐5	5280343
RB1	MOL006821	Epigallocatechin gallate	989‐51‐5	65064
RB1	MOL000006	Luteolin	491‐70‐3	5280445
RB1	MOL000098	Quercetin	117‐39‐5	5280343

### 3.18 Compound‐Drug Target‐Pathway Network Analysis

Figure 5I illustrates the intricate network of interactions between Triphala‐derived small molecules, their target genes, and ferroptosis‐related biological processes. Figure 5I shows the network, organized with chemical compounds (yellow nodes) at the bottom, connecting to their respective drug targets (blue nodes) in the middle, which in turn regulate various biological pathways and processes (red nodes) at the top. The visualization reveals that epigallocatechin gallate exhibits the most extensive connectivity, targeting multiple genes including GSTA1, MGST1, PPARG, and MAPK8, thereby influencing various ferroptosis‐related processes such as glutathione transferase activity and positive regulation of apoptotic process. Quercetin demonstrates similar versatility, targeting MAPK8, EGFR, MGST1, and GSTA1, modulating cellular responses to ROS, glutathione metabolism, and apoptotic processes. Ellagic acid specifically targets MAPK8 and PPARG to influence apoptosis regulation, while gallic acid targets PPARG and MAPK8 for apoptosis regulation and EGFR for ERK cascade regulation. Kaempferol affects glutathione and xenobiotic metabolism through GSTA1, and luteolin modulates cellular responses to ROS via MAPK8 and EGFR while affecting glutathione peroxidase activity through GSTA1.

### 3.19 Drug Sensitivity Analysis of Functional Genes

Table 7 presents the significant correlations between the eight functional genes and therapeutic compounds, revealing potential drug sensitivity patterns in oral cancer. Each gene demonstrates both positive and negative correlations with specific drugs. AKR1C3 strongly correlates with Elephantin_1835 (*r* = 0.62, *p* = 2.06E‐34) and negatively with the PLK inhibitor BI‐2536 (*r* = −0.22, *p* = 0.000112), which affects cell cycle regulation. CA9 shows a positive correlation with the PI3K inhibitor AZD6482 (*r* = 0.28, *p* = 5.83E‐07) and a negative correlation with the apoptosis regulator Sepantronium bromide (*r* = −0.36, *p* = 6.96E‐11). EGFR positively correlates with the apoptosis regulator Venetoclax (*r* = 0.32, *p* = 5.44E‐09) while showing a strong negative correlation with the EGFR‐specific inhibitor Osimertinib (*r* = −0.49, *p* = 2.5E‐20). GSTA1 and PPARG both demonstrate strong positive correlations with the BCL inhibitor Obatoclax Mesylate (*r* = 0.49, *p* = 3.09E‐20 and *r* = 0.53, *p* = 6.14E‐24, respectively), which regulates apoptosis. MAPK8 shows opposite correlations with two RTK signaling modulators: positive with SB505124 (*r* = 0.39, *p* = 1.12E‐12) and strongly negative with PD173074 (r = −0.68, *p* = 3.83E‐43). MGST1 positively correlates with ERK_2440 (*r* = 0.35, *p* = 3E‐10) affecting ERK MAPK signaling, while RB1 shows a negative correlation with LY2109761 (*r* = −0.65, *p* = 5.71E‐38). These findings suggest potential targeted therapeutic approaches for oral cancer treatment through specific drug combinations that modulate the identified functional genes.

**TABLE 7:** Drugs significantly correlated with functional genes. Correlation coefficients, *p*‐values, and pathway classifications for drugs showing significant positive or negative correlations with each of the eight functional genes.

Gene	Drug	Cor	*p*	Drug_pathway
AKR1C3	Elephantin_1835	0.621524	2.06E‐34	Unclassified
AKR1C3	BI‐2536_1086	−0.21799	0.000112	Cell cycle
CA9	AZD6482_2169	0.279697	5.83E‐07	PI3K/MTOR signaling
CA9	Sepantronium bromide_1941	−0.35995	6.96E‐11	Apoptosis regulation
EGFR	Venetoclax_1909	0.324149	5.44E‐09	Apoptosis regulation
EGFR	Osimertinib_1919	−0.493	2.5E‐20	EGFR signaling
GSTA1	Obatoclax Mesylate_1068	0.491939	3.09E‐20	Apoptosis regulation
GSTA1	OSI‐027_1594	−0.35443	1.41E‐10	PI3K/MTOR signaling
MAPK8	SB505124_1194	0.390156	1.12E‐12	RTK signaling
MAPK8	PD173074_1049	−0.67921	3.83E‐43	RTK signaling
MGST1	ERK_2440_1713	0.348435	3E‐10	ERK MAPK signaling
MGST1	OSI‐027_1594	−0.15737	0.005565	PI3K/MTOR signaling
PPARG	Obatoclax Mesylate_1068	0.531604	6.14E‐24	Apoptosis regulation
PPARG	Axitinib_1021	−0.41498	2.72E‐14	RTK signaling
RB1	SB505124_1194	0.218164	0.000111	RTK signaling
RB1	LY2109761_1852	−0.64656	5.71E‐38	Other

### 3.20 Molecular Docking Validation of Compound‐Target Interactions

Molecular docking simulations were conducted to validate the predicted interactions between Triphala‐derived bioactive compounds and six of the eight identified functional genes (AKR1C3, EGFR, GSTA1, MAPK8, MGST1, PPARG, and RB1). The docking results demonstrated strong binding affinities between the compounds and their respective target proteins, with binding energies ranging from −5.3 to −9.7 kcal/mol (Table 8). AKR1C3 showed the strongest binding affinity with kaempferol (−9.5 kcal/mol), indicating a highly favorable interaction. The docking pose revealed that kaempferol formed multiple hydrogen bonds with key amino acid residues in the active site of AKR1C3, contributing to the stability of the compound‐protein complex. EGFR demonstrated variable binding affinities with different Triphala compounds. Quercetin exhibited the highest binding energy (−9.7 kcal/mol) with EGFR, followed by epigallocatechin gallate (−9.3 kcal/mol), luteolin (−7.9 kcal/mol), and quinine (−7.4 kcal/mol). The molecular interactions showed that these compounds occupied the ATP‐binding pocket of EGFR, suggesting potential competitive inhibition mechanisms. GSTA1 showed strong binding with ellagic acid (−9.6 kcal/mol), with the compound fitting well into the glutathione‐binding site of the enzyme. This interaction pattern supports the predicted role of ellagic acid in modulating glutathione metabolism pathways related to ferroptosis. MAPK8 demonstrated moderate binding affinities with epigallocatechin gallate and kaempferol (both −7.3 kcal/mol). The docking poses revealed that both compounds interacted with the ATP‐binding domain of MAPK8, potentially affecting its kinase activity and downstream signaling pathways. MGST1 exhibited the weakest binding affinity among all tested proteins, with gallic acid showing a binding energy of −5.3 kcal/mol. Despite the relatively lower binding energy, the interaction pattern indicated specific binding to the active site of MGST1. PPARG showed strong interactions with multiple compounds, with quercetin demonstrating the highest binding affinity (−8.4 kcal/mol), followed by epigallocatechin gallate (−7.5 kcal/mol), luteolin (−6.7 kcal/mol), and kaempferol (−6.5 kcal/mol). These compounds bound to the ligand‐binding domain of PPARG, suggesting potential modulation of its transcriptional activity. RB1 exhibited strong binding affinities with epigallocatechin gallate (−9.5 kcal/mol), quercetin (−8.4 kcal/mol), and luteolin (−8.1 kcal/mol). The molecular interactions involved key residues in the pocket domain of RB1, potentially affecting its tumor suppressor functions.

**TABLE 8:** Molecular docking results of Triphala compounds with functional gene targets.

Target	MolName	Binding energy (kcal/mol)	Results
AKR1C3 (4dbs)	Kaempferol	−9.5	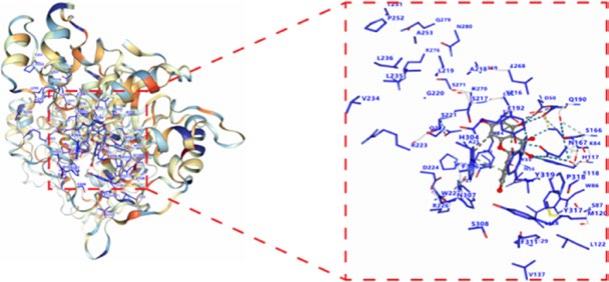
EGFR (1wt5)	Luteolin	−7.9	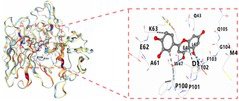
	Quinine	−7.4	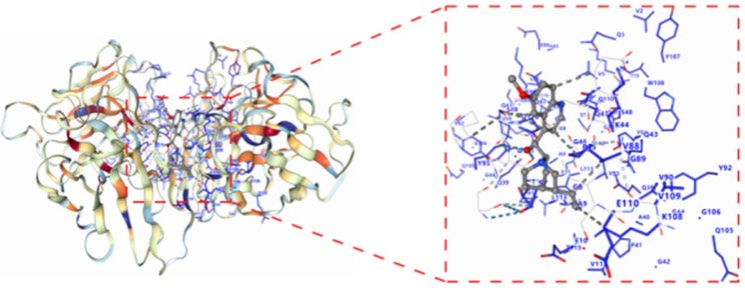
	Quercetin	−9.7	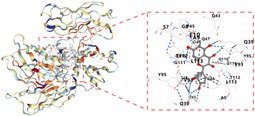
	Epigallocatechin gallate	−9.3	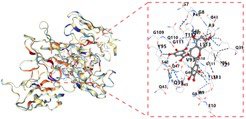
GSTA1 (7bib)	Ellagic acid	−9.6	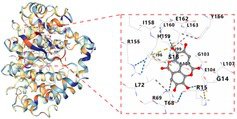
MAPK8 (4g1w)	Epigallocatechin gallate	−7.3	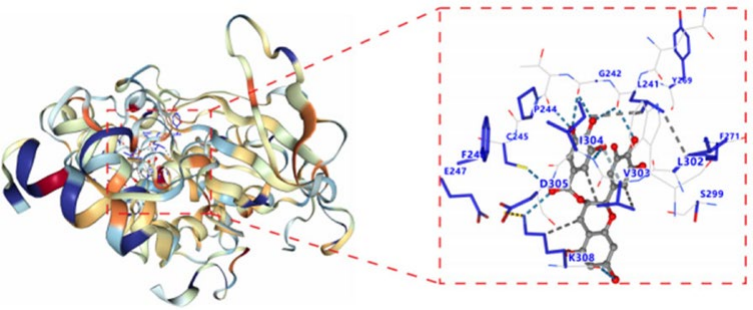
	Kaempferol	−7.3	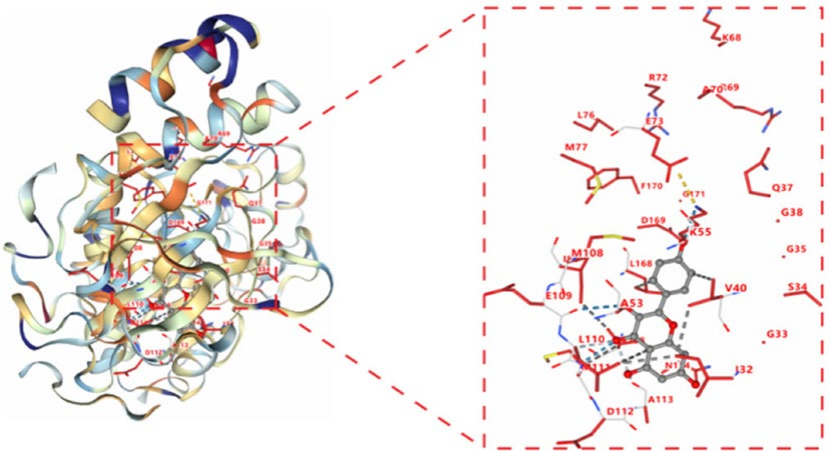
MGST1 (6vl4)	Gallic acid	−5.3	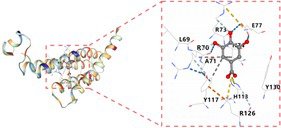
PPARG (9ck0)	Kaempferol	−6.5	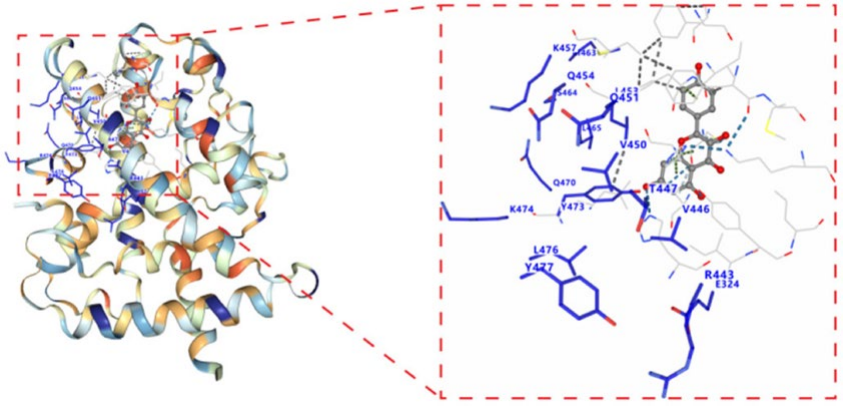
	Luteolin	−6.7	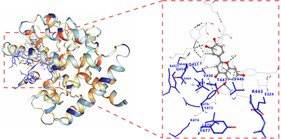
	Epigallocatechin gallate	−7.5	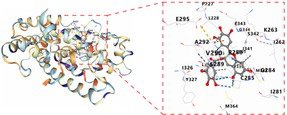
	Quercetin	−8.4	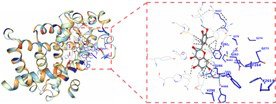
RB1 (9mml)	Epigallocatechin gallate	−9.5	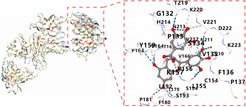
	Luteolin	−8.1	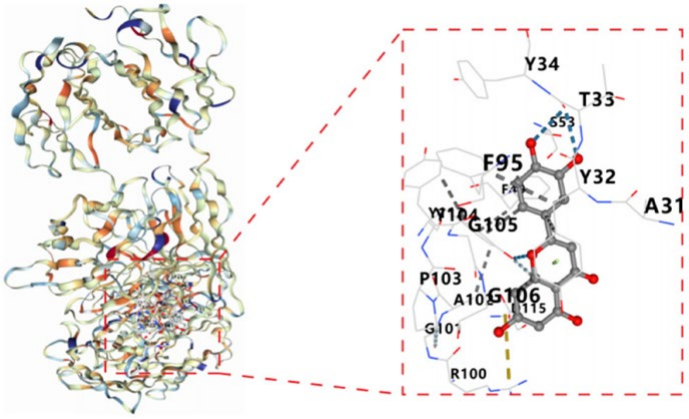
	Quercetin	−8.4	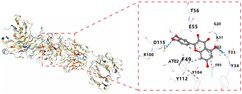

It should be emphasized, however, that these molecular docking results should be interpreted with caution. Docking simulations provide computational predictions of potential binding interactions based on estimated binding energies and geometric complementarity, and therefore indicate the likelihood and possible modes of compound–target binding rather than confirming that such interactions occur under physiological conditions. The favorable binding energies observed here should thus be regarded as supportive computational evidence that prioritizes candidate compound–target pairs for further study, not as direct proof of functional binding or inhibition. Consequently, the proposed interactions—and the competitive or modulatory mechanisms inferred from the docking poses—require confirmation by dedicated experimental approaches, such as surface plasmon resonance, isothermal titration calorimetry, or site‐directed mutagenesis in future work.

### 3.21 Triphala Extract Inhibits Oral Cancer Cell Viability in a Dose‐Dependent Manner

**FIGURE 6:**
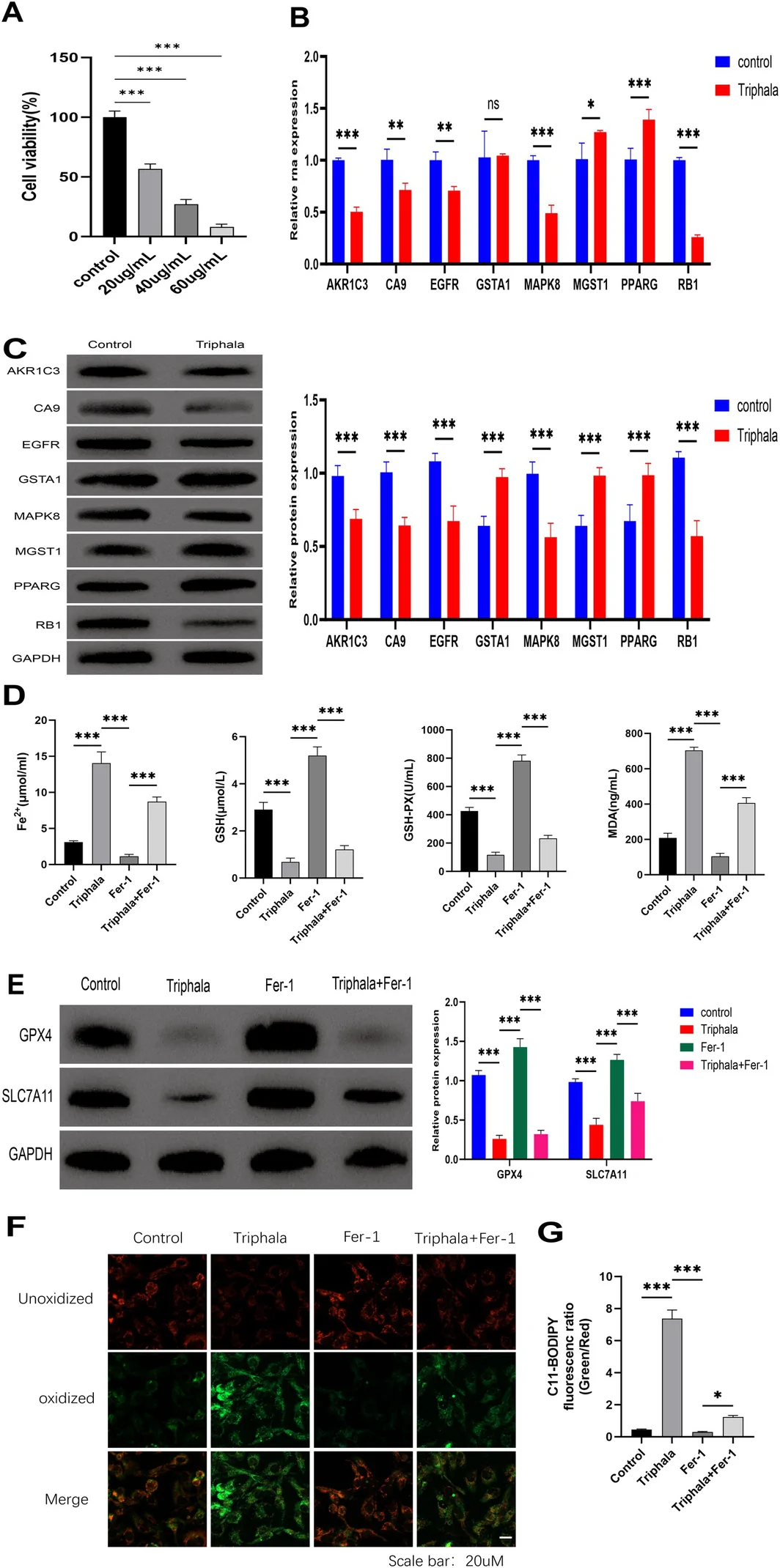
Triphala induces ferroptosis in oral cancer cells through modulation of ferroptosis‐related gene expression and lipid peroxidation. (A) Cell viability of CAL‐27 cells treated with various concentrations of Triphala extract (0, 20, 40, and 60 μg/mL) for 24 h was assessed by CCK‐8 assay. Data are presented as mean ± SD (*n* = 6). ***p < 0.001 versus control group. (B) Relative mRNA expression levels of eight ferroptosis‐related genes (AKR1C3, CA9, EGFR, GSTA1, MAPK8, MGST1, PPARG, and RB1) in CAL‐27 cells treated with vehicle (control) or 40 μg/mL Triphala extract for 24 h, detected by qRT‐PCR. GAPDH was used as an internal reference gene. Data are presented as mean ± SD (*n* = 6). ns, not significant; *p < 0.05, **p < 0.01, and ***p < 0.001 versus the control group. (C) Protein expression levels of eight ferroptosis‐related genes (AKR1C3, CA9, EGFR, GSTA1, MAPK8, MGST1, PPARG, and RB1) in CAL‐27 cells treated with vehicle (control) or 40 μg/mL Triphala extract for 24 h, detected by Western blot. GAPDH was used as a loading control. Representative blots from six independent experiments are shown. (D) Quantification of ferroptosis biomarkers including intracellular Fe^2+^ levels, GSH content, GSH‐Px activity, and MDA levels in CAL‐27 cells treated with vehicle (control), 40 μg/mL Triphala extract, 1 μM Fer‐1, or Triphala + Fer‐1 for 24 h, measured by ELISA. Data are presented as mean ± SD (*n* = 6). ***p < 0.001. (E) Protein expression levels of GPX4 and SLC7A11 in CAL‐27 cells treated with vehicle (control), 40 μg/mL Triphala extract, 1 μM Fer‐1, or Triphala + Fer‐1 for 24 h, detected by Western blot. GAPDH was used as a loading control. Representative blots from six independent experiments are shown. (F) Representative fluorescence images of lipid peroxidation in CAL‐27 cells treated with vehicle (control), 40 μg/mL Triphala extract, 1 μM Fer‐1, or Triphala + Fer‐1 for 24 h, stained with C11‐BODIPY 581/591 probe. Images show unoxidized lipids (red fluorescence, top row), oxidized lipids (green fluorescence, middle row), and merged images (bottom row) displaying the overlay of red and green channels. Red fluorescence indicates reduced lipids, and green fluorescence indicates oxidized lipids. Scale bar = 20 μM. (G) Quantification of C11‐BODIPY fluorescence ratio (green/red) from panel F. Data are presented as mean ± SD (*n* = 6). *p < 0.05, ***p < 0.001.

To evaluate the cytotoxic effect of Triphala extract on oral cancer cells, CAL‐27 cells were treated with increasing concentrations of Triphala (0, 20, 40, and 60 μg/mL) for 24 h, and cell viability was assessed by CCK‐8 assay. As shown in Figure 6A, Triphala extract significantly inhibited cell viability in a dose‐dependent manner (***p < 0.001). Compared to the control group (100% viability), treatment with 20 μg/mL Triphala reduced cell viability to 56.54%, while 40 and 60 μg/mL treatments further decreased viability to 27.06% and 8.031%, respectively. These results demonstrate that Triphala extract exerts potent cytotoxic effects on oral cancer cells, with the 20 μg/mL concentration selected for subsequent experiments based on its significant inhibitory effect while maintaining sufficient viable cells for further mechanistic studies.

### 3.22 Validation of Key Ferroptosis‐Related Genes by RT‐qPCR

To validate the expression patterns identified through network pharmacology analysis, RT‐qPCR was performed to assess the mRNA levels of eight core ferroptosis‐related genes following Triphala treatment (Figure 6B). Compared to the control group, Triphala treatment significantly downregulated the expression of AKR1C3, CA9, EGFR, MAPK8, and RB1 (*p* < 0.01 or *p* < 0.001). Among these, RB1 demonstrated the most pronounced downregulation, with relative expression reduced to approximately 0.25‐fold of the control level. Conversely, MGST1 and PPARG exhibited increased expression following Triphala treatment (*p* < 0.05 and *p* < 0.001, respectively), indicating potential protective mechanisms against oxidative stress and lipid peroxidation. GSTA1 showed no significant change in expression between control and Triphala‐treated groups (ns, *p* > 0.05).

### 3.23 Protein Expression Validation by Western Blot Analysis

Western blot analysis was performed to validate the protein expression levels of eight ferroptosis‐related genes following Triphala treatment (Figure 6C; uncropped blot images are provided in File S1). GAPDH was used as the internal reference control. Triphala treatment significantly downregulated the protein levels of AKR1C3, CA9, EGFR, MAPK8, and RB1 compared to the control group (*p* < 0.001). Among these, MAPK8 and RB1 showed the most pronounced reduction, with relative protein expression decreased to approximately 0.55‐fold of the control level. AKR1C3, CA9, and EGFR also demonstrated substantial downregulation. Conversely, GSTA1, MGST1, and PPARG protein expression were significantly upregulated following Triphala treatment (*p* < 0.001). GSTA1 and PPARG showed moderate increases to approximately 0.95–1.0‐fold, while MGST1 expression remained relatively stable or slightly elevated compared to control.

### 3.24 Triphala Induces Ferroptosis in Oral Cancer Cells Through Modulation of Iron Metabolism and Lipid Peroxidation

To confirm whether Triphala‐induced cell death is mediated through ferroptosis, we examined key ferroptosis biomarkers including intracellular Fe^2+^ levels, GSH content, GSH‐Px activity, and MDA accumulation (Figure 6D).

Triphala treatment significantly elevated both intracellular Fe^2+^ levels and MDA content compared to the control group (***p < 0.001), indicating pronounced iron accumulation and enhanced lipid peroxidation in oral cancer cells (Figure 6D). The ferroptosis inhibitor Fer‐1 treatment significantly reduced both Fe^2+^ levels and MDA content compared to the Triphala group (***p < 0.001), demonstrating that Fer‐1 can effectively suppress Triphala‐induced iron accumulation and lipid peroxidation. However, the rescue group (Triphala + Fer‐1) exhibited significantly elevated Fe^2+^ levels and MDA content compared to the Fer‐1 group (***p < 0.001), suggesting that Triphala partially overcomes the protective effect of Fer‐1 when used in combination.

Triphala treatment significantly decreased both GSH levels and GSH‐PX activity compared to the control group (***p < 0.001), indicating severe depletion of the antioxidant defense system in oral cancer cells (Figure 6D). The ferroptosis inhibitor Fer‐1 treatment significantly elevated both GSH levels and GSH‐PX activity compared to the Triphala group (***p < 0.001), demonstrating that Fer‐1 can effectively restore the antioxidant capacity. The rescue group (Triphala + Fer‐1) showed significantly increased GSH levels and GSH‐PX activity compared to the Fer‐1 group (***p < 0.001), suggesting partial restoration of the antioxidant defense system when Fer‐1 is co‐administered with Triphala.

### 3.25 Effects of Triphala and Ferroptosis Inhibitor Fer‐1 on GPX4 and SLC7A11 Protein Expression

Western blot analysis revealed that Triphala treatment significantly downregulated the protein expression of both GPX4 and SLC7A11 compared to the control group (*p* < 0.001) (Figure 6E). The ferroptosis inhibitor Fer‐1 alone markedly upregulated GPX4 and SLC7A11 expression compared to the Triphala group (*p* < 0.001). Notably, co‐treatment with Triphala and Fer‐1 significantly reversed the Fer‐1‐induced upregulation, reducing GPX4 and SLC7A11 expression compared to Fer‐1 alone (*p* < 0.001).

### 3.26 Triphala Induces Lipid Peroxidation Detected by C11‐BODIPY Fluorescence Imaging

To directly visualize lipid peroxidation in living cells, we employed the C11‐BODIPY 581/591 probe, which shifts from red to green fluorescence upon oxidation (Figure 6F). Quantitative analysis of the green/red fluorescence ratio (Figure 6G) confirmed these observations. Triphala treatment significantly elevated the C11‐BODIPY fluorescence ratio compared to the control group (***p < 0.001), reflecting severe lipid peroxidation. The ferroptosis inhibitor Fer‐1 treatment significantly reduced the fluorescence ratio compared to the Triphala group (***p < 0.001), demonstrating that Fer‐1 can effectively suppress Triphala‐induced lipid peroxidation. However, the rescue group (Triphala + Fer‐1) exhibited a significantly increased fluorescence ratio compared to the Fer‐1 group (*p < 0.05), suggesting that Triphala partially overcomes the protective effect of Fer‐1 when used in combination.

## 4 Discussion

Our experimental findings demonstrate that Triphala extract exerts potent cytotoxic effects on oral cancer cells through induction of ferroptosis, as evidenced by modulation of key ferroptosis regulatory proteins and biomarkers. The dose‐dependent inhibition of CAL‐27 cell viability, with significant reduction to 27.06% at 40 μg/mL, aligns with previous studies showing Triphala's selective cytotoxicity against various cancer cell lines while sparing normal cells (Sandhya et al. 2006). This selective anticancer property has been attributed to Triphala's ability to evoke differential intracellular ROS responses between normal and tumor cells, a phenomenon that appears central to its therapeutic mechanism. Our Western blot analysis revealed that Triphala treatment significantly downregulated both GPX4 and SLC7A11 protein expression, two critical components of the primary ferroptosis defense system. The SLC7A11‐GSH‐GPX4 axis represents the major antioxidant defense mechanism against ferroptosis, where SLC7A11 facilitates cystine uptake for GSH biosynthesis, and GPX4 utilizes GSH to reduce toxic lipid peroxides at cellular membranes (Wang et al. 2024). The concurrent suppression of both proteins by Triphala suggests a coordinated disruption of this protective axis, rendering cancer cells vulnerable to ferroptotic cell death. Notably, even in the presence of the ferroptosis inhibitor Fer‐1, which significantly upregulated GPX4 and SLC7A11 expression compared to Triphala alone, co‐treatment with Triphala and Fer‐1 markedly reversed this protective upregulation. This observation indicates that Triphala's ferroptosis‐inducing capacity is robust and can partially overcome pharmacological resistance mechanisms, a finding with significant therapeutic implications. Previous studies have established that downregulation of SLC7A11 expression enhances tumor cell sensitivity to ferroptosis and can synergize with immune checkpoint blockade therapy (Jiang and Sun 2024). The ability of Triphala to persistently suppress these key ferroptosis regulators, even in the presence of ferroptosis inhibitors, suggests its potential as a promising ferroptosis‐inducing agent for cancer therapy. Importantly, these experimental observations do not stand alone but represent the convergent endpoint of our multi‐layered analytical framework. Integrating these findings yields a coherent causal chain: Triphala‐derived compounds are predicted and docked to bind ferroptosis‐related targets (e.g., EGFR, AKR1C3, GSTA1, MGST1, PPARG); among these, MAPK8, MGST1, and PPARG are supported by genetic causal evidence (MR with colocalization); and, consistent with these predictions, Triphala experimentally suppresses the SLC7A11–GSH–GPX4 axis and elevates Fe^2+^, MDA, and lipid peroxidation, with rescue by ferrostatin‐1 confirming that the observed phenotype is ferroptosis‐dependent. Thus, computational prioritization, genetic causal inference, and experimental validation converge on the same mechanistic conclusion, providing a logically integrated basis for the gene‐ and pathway‐level discussion that follows.

The eight genes constituting our prognostic signature—AKR1C3, CA9, EGFR, GSTA1, MAPK8, MGST1, PPARG, and RB1—are mechanistically linked to both ferroptosis regulation and oral/head‐and‐neck carcinogenesis, which supports their biological plausibility as mediators of Triphala's ferroptosis‐related antitumor effects. Several of them act as endogenous suppressors of ferroptosis by reinforcing the glutathione‐dependent antioxidant defense that Triphala was found to disrupt in the present study. GSTA1, an alpha‐class glutathione S‐transferase, limits lipid peroxidation and suppresses ferroptosis while conferring sorafenib resistance in hepatocellular carcinoma through a GSTA1/CTNNB1 axis (Ma et al. 2024). Similarly, MGST1 protects tumor cells from ferroptosis by binding arachidonate 5‐lipoxygenase (ALOX5) and lowering lipid‐peroxide generation, thereby driving chemoresistance (Zhang et al. 2023), and AKR1C3 inhibits ferroptosis and enhances radio‐ and chemo‐resistance across multiple tumors by preserving GPX4 via HSPA5 (Ju et al. 2025). CA9, a hypoxia‐inducible enzyme, is strongly associated with poor vascularization, treatment resistance, and unfavorable prognosis in head and neck squamous cell carcinoma (Koukourakis et al. 2001), and its upregulation has recently been implicated in blocking ferroptosis in this tumor type (Fan et al. 2025). PPARG, a nuclear receptor governing lipid metabolism, modulates ferroptosis sensitivity through control of polyunsaturated‐fatty‐acid handling (e.g., ACSL4) and can itself drive ferroptosis in a context‐dependent manner (Han et al. 2021). The remaining genes couple ferroptosis to core oncogenic signaling: EGFR is frequently amplified and overexpressed in oral squamous cell carcinoma and represents its principal validated therapeutic target (Dai et al. 2014), and it also confers ferroptosis resistance such that EGFR inhibition sensitizes HNSCC cells to ferroptosis inducers (Liu et al. 2022); MAPK8 (JNK1) transduces oxidative‐stress signals that promote lipid‐peroxidation‐driven ferroptosis (Wang, Tan, et al. 2023). Importantly, EGFR and MAPK8 are not independent nodes but lie on a shared receptor‐tyrosine‐kinase–MAPK axis: EGFR activation propagates through the mitogen‐activated protein kinase cascade, of which JNK/MAPK8 is a stress‐responsive branch, and this cascade is tightly coupled to intracellular ROS and redox homeostasis (Wang, Tan, et al. 2023). Because a high‐ROS environment activates MAPK signaling to promote ferroptosis, whereas sustained EGFR signaling reinforces antioxidant capacity and thereby restrains lipid‐peroxidation‐driven death, the EGFR–MAPK8 relationship may function as a rheostat that sets the threshold for ferroptosis in oral cancer cells (An et al. 2024). Within this framework, Triphala‐induced suppression of EGFR together with modulation of MAPK8 would be expected to lower the antioxidant threshold and amplify ROS‐ and lipid‐peroxide‐mediated ferroptosis, providing a coherent signaling link between these two hub genes and the ferroptotic phenotype we observed. The tumor suppressor RB1 modulates ferroptosis, as Rb‐deficient hepatocellular carcinoma cells undergo markedly enhanced ferroptotic death upon sorafenib exposure (Louandre et al. 2015). Notably, some of these hub genes have already surfaced as Triphala targets in earlier network‐pharmacology studies—EGFR ranked among the most highly connected Triphala targets in gynecological cancer (Zhao et al. 2018), and both EGFR and PPARG were recovered as multi‐compound Triphala targets in a separate analysis (Wang, Liu, et al. 2019)—yet none of these genes had previously been connected to Triphala's ferroptosis‐inducing activity in oral cancer, underscoring the novelty of the present work. Clinically, this eight‐gene signature integrates hypoxia (CA9), glutathione/redox metabolism (GSTA1, MGST1, AKR1C3), lipid metabolism (PPARG), and canonical proliferative and tumor‐suppressive signaling (EGFR, MAPK8, RB1) into a single risk model whose components include established prognostic biomarkers and druggable targets, suggesting that it may not only stratify patient outcome but also help identify those most likely to benefit from ferroptosis‐directed strategies or from combining Triphala‐derived compounds with existing therapies such as EGFR inhibitors.

Beyond the association‐level evidence from differential expression and prognostic modeling, our Mendelian randomization (MR) analysis, combined with colocalization, provided genetic support for a causal contribution of three signature genes—MAPK8, MGST1, and PPARG—to oral cancer. The mechanistic value of this finding derives from the design of MR itself: because it uses germline genetic variants (here, cis‐eQTLs) as instrumental variables for gene expression, and because genotype is fixed at conception and randomly allocated at meiosis, MR estimates are substantially less vulnerable to the reverse causation and environmental confounding that bias conventional observational associations (Lovegrove et al. 2024; Burgess et al. 2021). The addition of colocalization strengthens this inference by testing whether the eQTL signal and the disease‐association signal at each locus are driven by a shared causal variant rather than by two distinct variants in linkage disequilibrium, thereby reducing the risk of spurious causal attribution (Giambartolomei et al. 2014). A causal signal that survives both analyses therefore carries greater mechanistic weight than expression correlation alone, prioritizing MAPK8, MGST1, and PPARG as the most credible effectors among the eight signature genes. Interpreted in a biological context, these three genes converge on the redox and lipid‐metabolic machinery that governs ferroptosis: MGST1 detoxifies lipid peroxides and, by binding arachidonate 5‐lipoxygenase (ALOX5), suppresses ferroptotic lipid peroxidation (Zhang et al. 2023); PPARG regulates polyunsaturated‐fatty‐acid handling and thereby modulates ferroptosis susceptibility (Han et al. 2021); and MAPK8 (JNK1) transduces oxidative‐stress signals that promote lipid‐peroxidation‐driven cell death (Wang, Tan, et al. 2023). The convergence of genetically inferred causality with these ferroptosis‐related functions suggests that MAPK8, MGST1, and PPARG are not merely passenger genes correlated with disease, but may causally shape oral cancer through ferroptosis‐related redox and lipid‐metabolic pathways, making them particularly well‐grounded targets for Triphala‐based intervention. Nevertheless, MR reflects the cumulative effect of lifelong, genetically determined differences in gene expression rather than acute pharmacological modulation, and its validity rests on assumptions (e.g., absence of horizontal pleiotropy) that cannot be fully excluded; the causal relationships inferred here should therefore be confirmed by targeted functional and in vivo perturbation studies (Lovegrove et al. 2024).

Our ELISA and lipid peroxidation analyses provide direct biochemical evidence that Triphala induces ferroptotic cell death through characteristic hallmarks of this process: elevated intracellular Fe^2+^ levels, increased lipid peroxidation products (MDA), depletion of the antioxidant defense system (GSH and GSH‐Px), and accumulation of oxidized lipids visualized by C11‐BODIPY staining. Critically, these metabolic changes were accompanied by concordant downregulation of SLC7A11 and GPX4 at the protein level, linking the observed biochemical phenotype to a defined molecular axis. As the catalytic subunit of the cystine/glutamate antiporter system xc^−^, SLC7A11 mediates cystine uptake for glutathione biosynthesis; its suppression by Triphala restricts cysteine supply and accounts for the marked GSH depletion we observed, which in turn disables the GSH‐dependent enzyme GPX4. The significant elevation of Fe^2+^ levels following Triphala treatment is consistent with ferroptosis being fundamentally an iron‐dependent cell death process, where redox‐active ferrous iron participates in Fenton reactions to generate hydroxyl radicals that initiate lipid peroxidation cascades (Li et al. 2022). Iron accumulation in cancer cells can promote the peroxidation of PUFA‐containing phospholipids, leading to membrane damage and ultimately cell death, making iron metabolism a critical regulatory node in ferroptosis. The marked increase in MDA content, a well‐established biomarker of lipid peroxidation, directly reflects the accumulation of toxic lipid peroxides that characterize ferroptotic cell death (Zhao et al. 2021). MDA and other lipid peroxidation products such as 4‐hydroxynonenal (4‐HNE) have been widely used to monitor ferroptosis in cancer cells, and their elevation serves as definitive evidence of ongoing ferroptotic processes (Lei and Gan 2024). Concurrent with lipid peroxide accumulation, we observed severe depletion of both GSH levels and GSH‐Px activity in Triphala‐treated cells, indicating comprehensive collapse of the cellular antioxidant defense system. GSH depletion is a hallmark of ferroptosis, as it impairs the activity of GPX4, the key enzyme responsible for reducing lipid hydroperoxides to non‐toxic lipid alcohols (Uti et al. 2025). Thus, the coordinated suppression of the SLC7A11–GSH–GPX4 axis provides a coherent mechanistic explanation for how Triphala dismantles the ferroptosis defense system and drives the downstream accumulation of iron and lipid peroxides. The C11‐BODIPY fluorescence imaging, which shifts from red to green fluorescence upon lipid oxidation, provided visual confirmation of extensive lipid peroxidation in living cells, with Triphala‐treated cells showing dramatically increased green fluorescence indicative of oxidized lipids. The ferroptosis inhibitor Fer‐1 significantly attenuated these biochemical changes, confirming that Triphala‐induced cell death proceeds through the ferroptosis pathway. However, the rescue group (Triphala + Fer‐1) still exhibited significantly elevated ferroptotic markers compared to Fer‐1 alone, demonstrating that Triphala can partially overcome ferroptosis resistance mechanisms even in the presence of pharmacological inhibitors. This persistent ferroptosis‐inducing activity, coupled with the multi‐targeted suppression of ferroptosis defense systems at both protein and metabolic levels, positions Triphala as a promising natural compound for cancer therapy, particularly for tumors that have developed resistance to conventional apoptosis‐inducing treatments.

Figure 6 constitutes the experimental cornerstone of this study, and its findings warrant fuller mechanistic interpretation, particularly regarding the apparently divergent responses of the eight signature genes. Triphala downregulated AKR1C3, CA9, EGFR, MAPK8, and RB1 at both the mRNA and protein levels, whereas GSTA1, MGST1, and PPARG were upregulated. At first glance this is counterintuitive, because GSTA1, MGST1, and PPARG generally act as suppressors of ferroptosis; however, all three are established targets of the Nrf2‐driven antioxidant‐response program that cells mobilize under oxidative and electrophilic stress (Wang, Tan, et al. 2023). Their induction is therefore best interpreted as a compensatory, stress‐adaptive response to the severe lipid peroxidation imposed by Triphala rather than as evidence that ferroptosis was averted. Critically, this compensatory upregulation was insufficient to rescue the cells: the concurrent and dominant suppression of the core SLC7A11–GSH–GPX4 axis, together with the measured collapse of GSH and GSH‐Px and the accumulation of Fe^2+^, MDA, and oxidized lipids, shows that the net cellular outcome was ferroptotic death. This interpretation is consistent with the central role of the SLC7A11–GSH–GPX4 axis as the principal ferroptosis checkpoint, whose inactivation licenses lipid‐peroxidation‐driven death even in the presence of residual antioxidant capacity (Jiang et al. 2021). The value of Figure 6 further lies in its four‐arm design (Control, Triphala, Fer‐1, and Triphala + Fer‐1), which advances the evidence from correlation to causation: ferrostatin‐1 attenuated Triphala‐induced Fe^2+^ accumulation, GSH/GSH‐Px depletion, MDA elevation, and C11‐BODIPY oxidation, confirming that these changes are causally driven by lipid peroxidation, while the incomplete rescue—Triphala + Fer‐1 remained significantly shifted toward the ferroptotic state relative to Fer‐1 alone—indicates that Triphala engages ferroptosis at multiple nodes simultaneously and can partially override pharmacological inhibition. Such multi‐target engagement is a recognized advantage of natural formulations and may render the resulting ferroptosis less easily circumvented than that induced by single‐target synthetic inducers such as erastin or RSL3. Mechanistically, because ferroptosis is distinct from apoptosis, its induction can eliminate drug‐tolerant “persister” and therapy‐resistant tumor cells that survive through dependency on the GPX4 axis (Hangauer et al. 2017), and Triphala‐mediated SLC7A11 suppression—the same event through which interferon‐γ from CD8^+^ T cells sensitizes tumors to ferroptosis—suggests potential synergy with immune‐checkpoint blockade (Wang, Green, et al. 2019). Collectively, the results in Figure 6 establish coordinated collapse of the SLC7A11–GSH–GPX4 axis as the central, experimentally validated mechanism of Triphala's antitumor activity in oral cancer.

**FIGURE 7:**
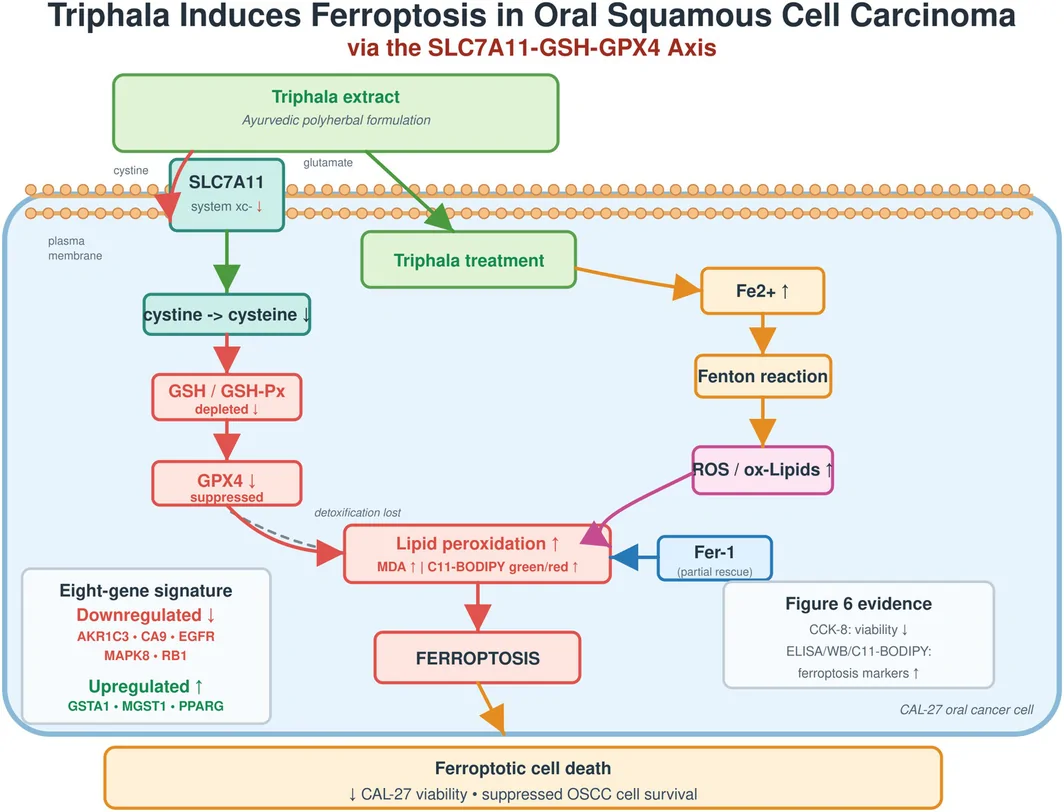
Schematic overview of the Triphala–ferroptosis–OSCC mechanistic axis. Triphala bioactive compounds inhibit SLC7A11 (system xc^−^), reducing cystine import and cysteine‐dependent glutathione (GSH) synthesis, which depletes GSH/GSH‐Px and inactivates GPX4. Concurrently, Triphala increases intracellular Fe^2+^, fueling the Fenton reaction and the accumulation of ROS and oxidized lipids. The combined collapse of the SLC7A11–GSH–GPX4 antioxidant axis and elevated oxidative stress promote lipid peroxidation (↑ MDA and ↑ C11‐BODIPY signal) and ferroptotic cell death, which is partially rescued by ferrostatin‐1 (Fer‐1). The eight‐gene signature is shown with genes downregulated (AKR1C3, CA9, EGFR, MAPK8, RB1) or upregulated (GSTA1, MGST1, PPARG) following Triphala treatment.

To integrate our computational and experimental findings into a unified storyline, we summarize the proposed Triphala–ferroptosis–OSCC mechanistic axis in Figure 7. Triphala‐derived bioactive compounds suppress the cystine transporter SLC7A11, thereby restricting cystine uptake and cysteine‐dependent GSH biosynthesis, depleting GSH/GSH‐Px, and inactivating GPX4, the central antioxidant defense against ferroptosis. In parallel, Triphala treatment promotes Fe^2+^ accumulation and Fenton‐reaction‐driven generation of ROS and oxidized lipids; together with the loss of GPX4‐mediated detoxification, this drives runaway lipid peroxidation (elevated MDA and C11‐BODIPY signal), culminating in ferroptotic death, which is partially reversed by ferrostatin‐1 (Fer‐1). The downregulated (AKR1C3, CA9, EGFR, MAPK8, RB1) and compensatorily upregulated (GSTA1, MGST1, PPARG) signature genes are overlaid on this axis, collectively linking Triphala treatment to suppressed CAL‐27 viability and OSCC cell survival.

Despite the comprehensive network pharmacology analysis and in vitro experimental validation presented in this study, several limitations should be acknowledged. First, our analysis relied heavily on publicly available databases (e.g., TCMSP, TCM Database@Taiwan, FerrDB, TCGA, and GEO) for compound, target, and gene expression information. The accuracy and completeness of these resources are inherently constrained by their curation criteria, potential annotation errors, and variable updating frequencies, and the ADME‐based screening thresholds (OB ≥ 30%, DL ≥ 0.18) may have excluded some genuinely active constituents while retaining others of limited in vivo relevance; the predicted compound–target relationships therefore require independent confirmation. Second, as a multi‐herb formulation, Triphala contains a large and chemically diverse array of bioactive constituents whose relative abundance, synergistic or antagonistic interactions, bioavailability, and in vivo metabolic transformation are difficult to capture fully by network pharmacology. Consequently, the specific compound or combination of compounds primarily responsible for the observed ferroptosis‐inducing effects cannot be definitively attributed from the present data. Third, our experimental validation was conducted exclusively in the CAL‐27 oral cancer cell line, and the findings require verification across a broader panel of oral cancer cell lines with diverse genetic backgrounds to establish the generalizability of Triphala's ferroptosis‐inducing effects. Fourth, the absence of in vivo animal experiments represents a significant limitation, as the pharmacokinetics, bioavailability, tissue distribution, and actual therapeutic efficacy of Triphala in living organisms remain unexplored, which are critical considerations for translating these findings into clinical applications. Fifth, while we identified eight key ferroptosis‐related genes through network pharmacology and validated their expression changes, the detailed molecular mechanisms underlying how specific bioactive compounds in Triphala interact with these targets and the downstream signaling cascades warrant further investigation, and the molecular docking results in particular remain computational predictions that await direct experimental confirmation. Sixth, the long‐term effects of Triphala treatment, potential development of resistance mechanisms, and interactions with the tumor microenvironment (Yang et al. 2026) and immune system in vivo have not been addressed in the current study. Future research incorporating additional cell lines, chemical standardization and component‐resolved analysis of the formulation, xenograft models, patient‐derived organoids, and clinical trials will be essential to fully elucidate Triphala's therapeutic potential and safety profile for oral cancer treatment.

## 5 Conclusion

This study demonstrates that Triphala exerts potent anticancer effects in oral cancer through induction of ferroptosis, as evidenced by comprehensive network pharmacology analysis and experimental validation. We identified and validated an eight‐gene prognostic signature (AKR1C3, CA9, EGFR, GSTA1, MAPK8, MGST1, PPARG, and RB1) that exhibits significant prognostic value and reveals the molecular mechanisms underlying Triphala's ferroptosis‐inducing capacity. Experimental validation confirmed that Triphala suppresses the SLC7A11‐GSH‐GPX4 axis, elevates intracellular Fe^2+^ levels and lipid peroxidation, and depletes antioxidant defenses, with these effects persisting even in the presence of ferroptosis inhibitors. The eight‐gene signature not only serves as a promising prognostic biomarker but also demonstrates significant correlations with immune cell infiltration and immunotherapy response, suggesting potential applications in combination therapeutic strategies. These findings bridge traditional herbal medicine with modern precision oncology, providing mechanistic insights and establishing a foundation for future translational research to develop Triphala‐based ferroptosis‐targeted therapies for oral cancer treatment.

## Author Contributions

**Linxin Jiang:** investigation, methodology, software, formal analysis, data curation. **Andreas Fichter:** project administration, supervision, writing – review and editing, resources, funding acquisition. **Yiwei Zhao:** conceptualization, investigation, methodology, validation, visualization, software, formal analysis, data curation, resources, writing – original draft, writing – review and editing. **Xianda Hu:** supervision, resources, project administration, writing – review and editing, investigation, funding acquisition. **Simin Li:** conceptualization, writing – original draft, writing – review and editing, investigation, methodology, validation, visualization, software, formal analysis, data curation, resources. **Gerhard Schmalz:** writing – review and editing, visualization, supervision, project administration, resources, funding acquisition. **Deborah Kreher:** investigation, methodology, data curation, writing – review and editing.

## Funding

The authors have nothing to report.

## Ethics Statement

This research does not involve animal experiments or human sample extraction, and therefore does not require ethical review. The study was conducted using publicly available data sources, without direct interaction with human or animal subjects, thus eliminating the need for ethical approval.

## Conflicts of Interest

The authors declare no conflicts of interest.

## Supporting information


**File S1:** Uncropped full‐length Western blot images for Figure 6C,E. All original, uncropped gel and blot images are presented. Left panels show bright‐field (white light) images displaying the protein ladder and membrane integrity; right panels show chemiluminescence images displaying the target protein bands. Three independent biological replicates (Rep 1–3) are shown for each target. Proteins detected: Figure 6C—AKR1C3, CA9, EGFR, GSTA1, MAPK8, MGST1, PPARG, RB1, and GAPDH (loading control); Figure 6E—GPX4, SLC7A11, and GAPDH (loading control).

## Data Availability

All datasets utilized in the study are publicly available. The codes during the current study are available from the corresponding author on reasonable request.
