# Supplementary material for: Triphala Targets the SLC7A11–GSH–GPX4 Axis to Trigger Ferroptosis in Oral Cancer: An Integrated Network Pharmacology, Molecular Docking, and Experimental Validation Study
Source: Chem Biol Drug Des. 2026 Aug 26;108(2):e70375. doi: 10.1111/cbdd.70375 (PMC13510106; doi:10.1111/cbdd.70375)
Supplement: Supplementary file 1 [file CBDD-108-e70375-s001.docx]

**Supplementary File S1.** Uncropped full-length Western blot images for Figure 6C and Figure 13E. All original, uncropped gel and blot images are presented. Left panels show bright-field (white light) images displaying the protein ladder and membrane integrity; right panels show chemiluminescence images displaying the target protein bands. Three independent biological replicates (Rep 1–3) are shown for each target. Proteins detected: Figure 6C — AKR1C3, CA9, EGFR, GSTA1, MAPK8, MGST1, PPARG, RB1, and GAPDH (loading control); Figure 6E — GPX4, SLC7A11, and GAPDH (loading control).

Figure 13C-AKR1C3

| **Three replications** | **bright-field (white light) images** | **chemiluminescence images** |
| --- | --- | --- |
| **Replication 1** |  |  |
| **Replication 2** |  |  |
| **Replication 3** |  |  |

Figure 13C-CA9

| **Three replications** | **bright-field (white light) images** | **chemiluminescence images** |
| --- | --- | --- |
| **Replication 1** |  |  |
| **Replication 2** |  |  |
| **Replication 3** |  |  |

Figure 13C-EGFR

| **Three replications** | **bright-field (white light) images** | **chemiluminescence images** |
| --- | --- | --- |
| **Replication 1** |  |  |
| **Replication 2** |  |  |
| **Replication 3** |  |  |

Figure 13C-GSTA1

| **Three replications** | **bright-field (white light) images** | **chemiluminescence images** |
| --- | --- | --- |
| **Replication 1** |  |  |
| **Replication 2** |  |  |
| **Replication 3** |  |  |

Figure 13C-MAPK8

| **Three replications** | **bright-field (white light) images** | **chemiluminescence images** |
| --- | --- | --- |
| **Replication 1** |  |  |
| **Replication 2** |  |  |
| **Replication 3** |  |  |

Figure 13C-MGST1

| **Three replications** | **bright-field (white light) images** | **chemiluminescence images** |
| --- | --- | --- |
| **Replication 1** |  |  |
| **Replication 2** |  |  |
| **Replication 3** |  |  |

Figure 13C-PPARG

| **Three replications** | **bright-field (white light) images** | **chemiluminescence images** |
| --- | --- | --- |
| **Replication 1** |  |  |
| **Replication 2** |  |  |
| **Replication 3** |  |  |

Figure 13C-RB1

| **Three replications** | **bright-field (white light) images** | **chemiluminescence images** |
| --- | --- | --- |
| **Replication 1** |  |  |
| **Replication 2** |  |  |
| **Replication 3** |  |  |

Figure 13C-GAPDH

| **Three replications** | **bright-field (white light) images** | **chemiluminescence images** |
| --- | --- | --- |
| **Replication 1** |  |  |
| **Replication 2** |  |  |
| **Replication 3** |  |  |

Figure 13E

Fig 13E-GPX4

| **Three replications** | **bright-field (white light) images** | **chemiluminescence images** |
| --- | --- | --- |
| **Replication 1** |  |  |
| **Replication 2** |  |  |
| **Replication 3** |  |  |

Fig 13E-SLC7A11

| **Three replications** | **bright-field (white light) images** | **chemiluminescence images** |
| --- | --- | --- |
| **Replication 1** |  |  |
| **Replication 2** |  |  |
| **Replication 3** |  |  |

Fig 13E-GAPDH

| **Three replications** | **bright-field (white light) images** | **chemiluminescence images** |
| --- | --- | --- |
| **Replication 1** |  |  |
| **Replication 2** |  |  |
| **Replication 3** |  |  |
